# BCL2 inhibition reveals a dendritic cell-specific immune checkpoint that controls tumor immunosurveillance

**DOI:** 10.1158/2159-8290.CD-22-1338

**Published:** 2023-08-25

**Authors:** Liwei Zhao, Peng Liu, Misha Mao, Shuai Zhang, Camille Bigenwald, Charles-Antoine Dutertre, Christian H. K. Lehmann, Hui Pan, Nicolas Paulhan, Lukas Amon, Aitziber Buqué, Takahiro Yamazaki, Lorenzo Galluzzi, Benoit Kloeckner, Aymeric Silvin, Yuhong Pan, Hui Chen, Ai-Ling Tian, Pierre Ly, Diana Dudziak, Laurence Zitvogel, Oliver Kepp, Guido Kroemer

**Affiliations:** 1 Centre de Recherche des Cordeliers, Equipe labellisée par la Ligue contre le cancer, Université de Paris, Sorbonne Université, Inserm U1138, Institut Universitaire de France, Paris, France; 2 Metabolomics and Cell Biology Platforms, Institut Gustave Roussy, Villejuif, France; 3 Faculté de Médecine, Université de Paris Saclay, Kremlin Bicêtre, France; 4 Surgical Oncology Department, Sir Run Run Shaw Hospital, Zhejiang University; 5 Department of Respiratory and Critical care Medicine, Union Hospital,Wuhan; 6 INSERM U1015, Equipe Labellisée - Ligue Nationale contre le Cancer, Villejuif, France; 7 Gustave Roussy Cancer Campus, Villejuif Cedex, France; 8 Center of Clinical Investigations in Biotherapies of Cancer (CICBT) 1428, Villejuif, France; 9 Laboratory of Dendritic Cell Biology, Department of Dermatology, University Hospital of Erlangen, Friedrich-Alexander-University Erlangen-Nürnberg, Erlangen, Germany; 10 Medical Immunology Campus Erlangen (MICE), Erlangen, Germany; 11 Deutsches Zentrum für Immuntherapie (DZI), Erlangen, Germany; 12 Comprehensive Cancer Center Erlangen - European Metropolitan Area of Nuremberg, Erlangen, Germany; 13 Department of Radiation Oncology, Weill Cornell Medical College, New York, NY, USA; 14 Sandra and Edward Meyer Cancer Center, New York, NY, USA; 15 Caryl and Israel Englander Institute for Precision Medicine, New York, NY, USA; 16 Institut du Cancer Paris CARPEM, Department of Biology, Hôpital Européen Georges Pompidou, AP-HP, Paris, France

**Keywords:** cellular immunity, immune checkpoint inhibition, non-small cell lung cancer, PD-1 blockade, sarcoma

## Abstract

We developed a phenotypic screening platform for the functional exploration of dendritic cells (DC). Here, we report a genome-wide CRISPR screen that revealed BCL2 as an endogenous inhibitor of DC function. Knockout of BCL2 enhanced DC antigen presentation and activation as well as the capacity of DCs to control tumors and to synergize with PD-1 blockade. The pharmacological BCL2 inhibitors venetoclax and navitoclax phenocopied these effects and caused a cDC1-dependent regression of orthotopic lung cancers and fibrosarcomas. Thus, solid tumors failed to respond to Bcl2 inhibition in mice constitutively devoid of cDC1, and this was reversed by the infusion of DCs. Moreover, cDC1 depletion reduced the therapeutic efficacy of BCL2 inhibitors alone or in combination with PD-1 blockade and treatment with venetoclax caused cDC1 activation, both in mice and in patients. In conclusion, genetic and pharmacological BCL2 inhibition unveils a DC-specific immune checkpoint that restrains tumor immunosurveillance.

## Introduction

Oncology of the 21^st^ century is marked by the ever-expanding number of targeted agents that act on specific oncoproteins or molecules operating downstream of such oncogene products. Such targeted agents exploit the ‘addiction’ of cancer cells to such pathways and are generally conceived to act in a cell-autonomous fashion (1). However, the most successful innovation in clinical oncology has been the development of immune checkpoint inhibitors (ICIs) targeting CTLA-4 or the PD-1/PD-L1 interaction across multiple different cancer types (2). The unprecedented success of these treatments demonstrates the possibility to unleash the immune system for cancer treatment.

Driven by this consideration, multiple groups have developed genome-wide genetic screening methods to identify novel druggable immune checkpoints. Such screens can be designed to identify genes the knockdown or knockout of which influences immunosurveillance. They can be performed on cancer cells to identify genes that confer resistance to T cell-mediated killing (3, 4), upregulate MHC class I molecules without upregulating PD-L1 (5) or downregulate immunosuppressive CD47 (6). Alternatively, such screens can be performed on immune cells, in particular on T lymphocytes, to identify genes the silencing of which enhances *in vitro* T cell proliferation (7) and improves cancer cell killing *in vitro* (8), stimulates the production of specific cytokines (7, 9), augments T cell infiltration of, and proliferation within, tumors (10), favors T cell persistence in tumors (11), or prevents T cell dysfunction in an immunosuppressive environment (12).

In contrast to T cells which are relatively abundant and can be driven into proliferation, dendritic cells (DCs) are relatively low-abundant and terminally differentiated, rendering genome-wide screens on primary DCs impractical. This collides with the cardinal importance that DCs play in the ignition of anticancer immune responses executed by T lymphocytes. For example, *Batf3*^-/-^ mice lacking a specific DC subset, the cDC1 cells, are unable to mount T cell responses against viral infections (13). Moreover, cancers evolving in *Batf3*^-/-^ mice do not respond to PD-1 blockade (14, 15) or other types of immunotherapy (16–18).

Here, we report the results of a genome-wide CRISPR/Cas9-based screen that involves two steps, (i) the genetic manipulation of immortal (and hence infinitely expandable) DC precursors, and (ii) the de-immortalization and differentiation of these cells to generate DCs that can be characterized *in vitro* and *in vivo*. We demonstrate that BCL2, an oncoprotein that is clinically targeted for the treatment of specific hematopoietic cancers, acts as a checkpoint to restrain the function of DCs with respect to tumor immunosurveillance. Thus, BCL2 inhibition has cDC1-dependent, T lymphocyte-mediated antineoplastic effects against solid cancers that do not respond to BCL2 inhibitors in an immunodeficient context.

## Results

### A genetic screen identifies BCL2 as an endogenous inhibitor of dendritic cell function

**Figure 1:**
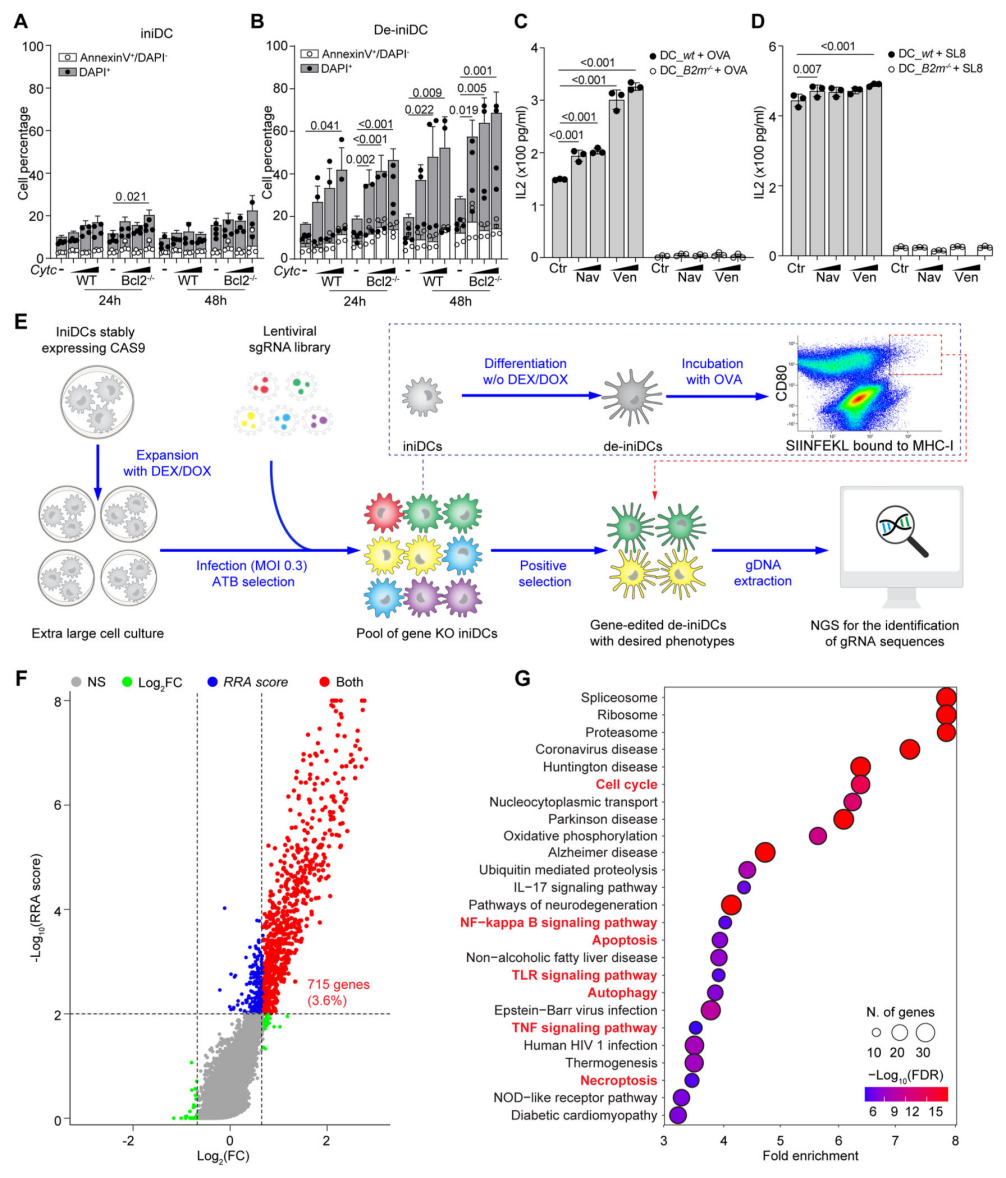
CRISPR KO screens for the identification of genes that act as DC immune checkpoint. **(A, B)** Inducible immortalized dendritic cell (iniDC) precursors and de-induced/differentiated immature DCs (de-iniDCs) were incubated with different concentrations of cytochrome c (*Cyt c*, 1, 2.5, 5 mg/mL) for 24 or 48 hours before being subjected to annexin V/DAPI staining. The percentages of apoptotic (annexin V^+^ DAPI^-^) and dead (DAPI^+^) cells are reported as stacked bar charts (mean ± SD with all data points). (**C,D**) Wild type (*wt*) or beta 2 microglobulin knockout (*B2m*^-/-^) de-iniDCs were treated with navitoclax (Nav), venetoclax (Ven), or DMSO control (Ctr) before incubated with ovalbumin (OVA) or OVA peptide (S257-L264, SL8) to stimulate the activation of specific TCR-expressing B3Z T cell hybridoma. The secretion of IL2 during this process was quantified to indicate antigen presentation capability and the results are reported as bar charts (mean ± SD with all data points). Statistical analysis in A-D was performed using Tukey’s multiple comparisons test. (**E**) The schematic pipeline of a pooled genome-wide CRISPR KO screen and the thresholds for the flow cytometric enrichment of cells with desired gain-of-function phenotype. Cas9-expressing iniDCs (iniDC_Cas9) were infected with a lentiviral CRISPR knockout library and following antibiotic selection, dexamethasone/doxycycline (Dex/Dox) were withdrawn to differentiate transduced cells into de-iniDCs, that were then exposed to OVA and subjected to flow cytometric enrichment of mature antigen-presenting DCs. The abundance of gRNA sequences in selected cells was quantified with next generation sequencing. (**F**) A volcano plot displays the abundance of gRNAs/gene comparing sorted with unsorted cells as fold change (FC) versus significance (RRA score calculated with MAGeCK software). As cutoffs log2FC > 1.6 and RRA score < 0.001 have been chosen. Genes were significantly different from controls for both parameters (red dots, 715 genes) were used to perform a KEGG pathway mapping (**G**) and the gene enrichment factor is shown for the indicated pathways (dot size indicates number of genes, and dot color represents estimated false discovery rate (FDR)).

Recently, we developed a protocol for CRISPR/Cas9-mediated gene knockout in conditionally immortalized immature dendritic cells (DCs), which can be limitlessly expanded before their differentiation/maturation (19). For this, we used an immature DC cell line from C57BL/6 mice, in which the SV40 large T cell antigen (SV40LgT) is expressed under the control of a Tet-on (doxycycline inducible) promoter and the reverse tetracycline transactivator is fused to the ligand-binding domain of a mutated glucocorticoid receptor (GR). Due to the dual blockade of retinoblastoma (RB) and tumor protein 53 (TP53) by SV40LgT, these cells are in an inducible/immortalized state (iniDCs) in the presence of doxycycline and the GR agonist dexamethasone, yet are de-induced/de-immortalized by the simultaneous removal of both factors (de-iniDCs) (20). In contrast to iniDCs, such de-iniDCs pinocytose extracellular proteins that reach their cytosol, meaning that (like cDC1 cells) (21), they become susceptible to apoptosis induction by addition of cytochrome *c* (Cyt *c*) to the culture media (Fig. 1A,B) and can present ovalbumin (OVA) protein to B3Z hybridoma cells expressing a transgenic T cell receptor recognizing the OVA-derived peptide SIINFEKL bound to H2-K^b^ MHC class I molecules (Fig. 1C,D).

IniDCs that were equipped with Cas9 were expanded by culture with DEX/DOX and then infected by a lentiviral library encoding bar-coded single guided RNAs (sgRNAs) that cover the entire mouse exome. The pool of sgRNA-expressing iniDCs then were differentiated into de-iniDCs, pulsed with OVA protein and subjected to several rounds of positive selection to purify those DCs that express high levels of the costimulatory ligand CD80 as well as SIINFEKL bound to H2-K^b^. This selection was possible due to immunostaining with antibodies recognizing CD80 and SIINFEKL/H2-K^b^ complexes, followed by cytofluorometric sorting of the double-positive cells. The sequences encoding the sgRNAs were amplified by PCR from the non-selected cell pool and sorted cells. The abundance of sgRNAs was quantified by next generation sequencing followed by data analysis using the MAGeCK-RRA program (22) for the identification of guidance RNAs (gRNA) associated with improved antigen presentation (Fig. 1E). A total of 715 genes (covering 3.6% of the genome-wide library) were significantly enriched in the sorted pool of cells that yielded gain-of-function phenotypes (Fig. 1F, Table S1). Of note, a significant fraction of the genes targeted by these gRNAs were connected to 7 cellular processes that are potentially druggable for cancer treatment such as apoptosis, autophagy, cell cycle, as well as the NF-κB, tumor necrosis factor (TNF) and toll-like receptor (TLR) signaling pathways (Fig. 1G).

**Figure 2:**
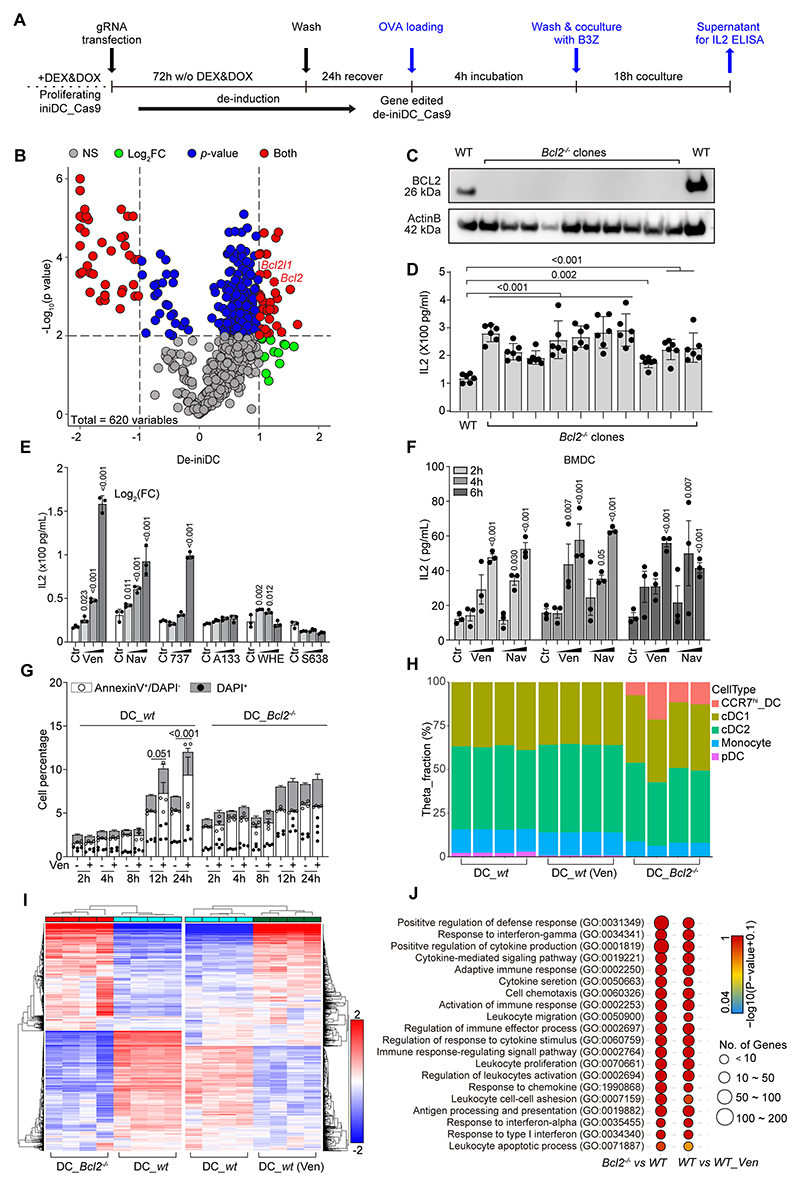
Arrayed CRISPR screening and validation of BCL2 as a target for DC stimulation (**A**) A scheme illustrates the arrayed CRISPR KO screening procedure. Cas9-expressing iniDCs (iniDC_Cas9) were transfected with individual gRNAs in the absence of Dex/Dox to establish specific gene KO and simultaneously differentiate the precursors into immature de-iniDC_Cas9, which were then exposed to OVA, washed and cocultured with B3Z T cell hybridoma cells that would be activated to secret interleukin 2 (IL2), indicating antigen cross-presentation capacity. (**B**) Log2 fold change (FC) of IL2 quantity for each KO as compared with non-target gRNA transfected controls and corresponding p-values (obtained with *t*-test) were used to generate a volcano plot. As cutoffs log2FC > 1 and p < 0.001 were chosen. gRNAs led to significant change of IL2 production from non-targeting control gRNA for both parameters are shown in red. Bcl2-targeting gRNA-transfected de-iniDC_Cas9 cells were cloned by flow cytometry and expanded. (**C, D**) Clones exhibiting Bcl2 protein levels undetectable by immunoblot (**C**) were evaluated for their capability to cross-present OVA to B3Z cells, as indicated by the secretion of IL2 (**D**). The significance of increased antigen cross-presentation over wild type (*wt*) de-iniDC_Cas9 cells were calculated by means of *t*-test. P-values are labelled in the figure as comparing between the indicated groups. (**E**) Differentiated iniDCs (de-iniDC) were treated with the indicated chemical inhibitors of Bcl2 family proteins, at different concentrations (1, 5, 10 μM), overnight before incubation with soluble OVA in the presence of the indicated treatments for 6 h. Venetoclax (Ven), navitoclax (Nav), and ABT737 are targeting Bcl2; A-1331852 and WEHI-539 are targeting Bcl-XL; S63845 is targeting Mcl1. The cells were washed and cocultured with B3Z T cells to evaluate their capability in priming the latter to secret IL2 as quantified by ELISA. (**F**)Treatments with Nav and Ven were applied to bone marrow-derived DCs (BMDCs) for 2~6 h before exposure to OVA to test their capability to activate B3Z cells. After treatment with venetoclax at the highest concentration, (**G**) De-iniDC viability was assessed by annexin V/DAPI staining and the percentage of apoptotic (annexin V^+^ DAPI^-^) and dead (DAPI^+^) cells is reported as stacked bar chart, mean ± SD with all data points. Statistical significance was calculated by means of two-way ANVOA with Dunnett’s multiple comparisons test. (**H**) Bulk RNASeq data obtained with wild type (*wt*) de-iniDC, Ven-treated *wt* de-iniDCs and *Bcl2*^-/-^ de-iniDCs was deconvoluted using the BayesPrism algorithm and aligned with scRNA-sequencing signatures from Brown et al (59). Barplot represents virtual proportions (Theta_Fraction) of scRNAseq DC signatures in bulk RNAseq samples. (**I**) Bulk RNASeq data also indicates a set of genes that are regulated in Ven-treated *wt* de-iniDCs and *Bcl2*^-/-^ de-iniDCs as compared to untreated *wt* de-iniDCs. (**J**) Based on the commonly upregulated genes, the GO term enrichment analysis for immune cell activation related genes is depicted as gene numbers with log transformed adjusted p values for these two comparisons.

In the next step, we manually selected 620 gRNAs falling into these 7 categories (Table S2). These gRNAs were individually transfected into Cas9-expressing iniDCs, followed by de-induction/de-immortalization, OVA loading of de-iniDCs, confrontation of the washed de-iniDCs with B3Z T cell hybridomas specific for H2-K^b^-bound SIINFEKL, and final measurement of interleukin-2 (IL2) production by B3Z cells (Fig. 2A). This screen yielded 34 gRNAs that significantly improved antigen presentation by de-iniDCs, among which we identified BCL2 as a prominent hit (Fig. 2B, Table S2). We subsequently generated multiple independent de-ini-DC clones lacking BCL2, all of which exhibited a gain-of-function phenotype with respect to the presentation of OVA-derived SIINFEKL to B3Z cells (Fig. 2C,D). Consistently, several KEGG pathways that cover the top hits from genome-wide screen (Fig. 1G) matched with the 34 targets that appear in the arrayed screen (Fig. S1A). These pathways included several apoptosis-relevant genes including those coding for BCL2 itself, the close BCL2 homolog BCLXL (*Bcl2l1*), BCL2 binding C-component 3 (*Bbc3*), three caspases (*Casp2*, *Casp3*, *Casp6*) and the catalytic subunit of calpain-1 (*Capn1*).

Altogether, the aforementioned results identify several apoptosis-relevant genes as candidate immune checkpoints acting at the level of DCs. We decided to focus on BCL2, because it is the sole target for which an FDA/EMA-approved drug is available for the treatment of specific blood cancers (in particular, acute myeloid leukemia, chronic lymphocytic leukemia and small lymphocytic leukemia) (23).

### Pharmacological BCL2 inhibition stimulates dendritic cell function

Addition of pharmacological inhibitors of BCL2 such as ABT737, navitoclax and venetoclax, (but not that of agents inhibiting other members of the BCL2 family such as A1331852, S63845 and WEHI539) enhanced antigen presentation by wild type (WT) de-iniDCs (Fig. 2E) and bone marrow-derived DCs (BMDCs) (Fig. 2F). BCL2 inhibitors can induce both apoptosis (which depends on BAX but not ATG7) and autophagy (which depends on ATG7 but not BAX) (24, 25). Accordingly, navitoclax and venetoclax induced caspase activation in WT and autophagy-deficient *Atg7*^-/-^ de-iniDCs (and less so in *Bcl-2*^-/-^ and *Bax*^-/-^ de-iniDCs), as well as the autophagy-associated LC3B lipidation giving rise to electrophoretically mobile LC3B-II in WT, *Bcl-2*^-/-^ and *Bax*^-/-^ (but not *Atg7*^-/-^) de-iniDCs (Fig. S1B,C). However, treatment of wild type (WT) de-iniDCs with 10 μM venetoclax only killed a fraction of the cells after a 24-hour treatment (~12%). No extra cell death was induced by venetoclax in *Bcl2*^-/-^ de-iniDCs. Moreover, there was no major increase in spontaneous cell death events in *Bcl2*^-/-^ de-iniDCs compared to WT de-iniDCs (Fig. 2G).

Navitoclax and venetoclax enhanced antigen presentation by WT de-iniDCs, but not by constitutively overactive *Bcl-2*^-/-^ de-iniDCs, in line with the idea that they indeed act on target. Both BCL2 inhibitors failed to enhance antigen presentation by autophagy-deficient (*Atg5*^-/-^ or *Atg7*^-/-^) de-iniDCs, but continued to act on apoptosis-deficient (*Bax*^-/-^) de-iniDCs, suggesting that they stimulate DC function through autophagy-related processes (but not apoptosis) (Fig. S1D,E), in line with prior reports that *Atg5*^-/-^ or *Atg7*^-/-^ DCs are deficient in antigen presentation (26, 27).

Bulk RNAseq revealed that the transcriptome of de-iniDCs globally resembles that of conventional DCs (cDCs), in particular cDC1 cells (Fig. 2H). In addition, the knockout of *Bcl2* caused de-iniDCs to acquire the signature of migratory cDC1s (which are CCR7^+^). *Bcl-2* KO and the clinically employed BCL2 inhibitor venetoclax upregulated a partially overlapping set of mRNAs in de-iniDCs (Fig. 2I) that represented multiple genes involved in the immune response including the activation of cytokine responses and improved antigen processing and presentation (Fig. 2I,J). Among the 79 genes that were strongly upregulated (by ≥2 fold) in de-iniDCs by both Bcl2 knockout and venetoclax treatment, a large majority (n=65, 82%) were connected to the Type 1 interferon response (Table S3). qRT-PCR confirmed that venetoclax treatment of WT cells as well as *Bcl2* knockout induced the upregulation of mRNAs encoding Type 1 interferons (*Ifna1*, *Ifnb1*), the two subunits of the common Type 1 interferon receptors (*Ifnar1*, *Ifnar2*) and typical downstream target of Type 1 interferon signaling (*Cxcl10*, *Isg15*, *Mx1*). Moreover, pharmacological or genetic inhibition of Bcl2 resulted in an increase of *Tmem173/Sting* mRNA and its downstream effector *Irf3* (Fig. S2A). Accordingly, after venetoclax treatment or *Bcl2* knockout, the abundance of cGAS protein, as well as the phosphorylation of STING and IRF3 increased (Fig. S2B,C). This points to the activation of the STING pathway. Indeed, the knockout of *Tmem173/Sting*, that of its downstream effector *Irf3*, as well as the knockout of *Ifnar1* or *Ifnar2* (which are non-redundant because they form heterodimers) abolished the capacity of venetoclax to stimulate antigen presentation by de-iniDCs (Fig. S2D). Accordingly, blockade of Ifnar1 protein with a monoclonal antibody blunted the capacity of venetoclax to activate antigen presentation by de-iniDCs (Fig. S2E). Moreover, in accord with prior records showing that BCL inhibitors can induce the release of mitochondrial DNA (mtDNA) from cells (28, 29), venetoclax treatment of WT de-iniDCs and *Bcl2* KO led to an increase in double-stranded DNA in the extramitochondrial cytoplasm (Fig.S2F,G).

*Bcl-2* KO de-ini-DC compared to WT controls also exhibited a higher secretion of the interleukins (IL) IL1β and IL6 (determined by ELISA), as well as higher expression of the chemokine receptors CCR2 and CXCR3, co-stimulatory receptors (CD80, CD83 and CD86) and activation markers (CD40, MHC class II molecules, PD-L1) than WT controls (Fig. S3A). These findings (except the upregulation of PD-L1) could be recapitulated by adding venetoclax to WT de-iniDCs and to a lesser degree by other pharmacological agents acting on the BCL2 family (Fig. S3A). Of note, neutralization of CD80 and CD86 both reduced antigen presentation of OVA to B3Z cells by navitoclax and venetoclax, suggesting that their upregulation might be functionally relevant (Fig. S3B,C).

In sum, the aforementioned results support the contention that pharmacological BCL2 inhibition can act on target to enhance the immunostimulatory potential of DCs. This effect is mediated via the activation of the type-1 interferon pathway and the upregulation of costimulatory receptors.

### BCL2 inhibitors activate cDC1 cells in mice and human

A prior report published in *Cancer Discovery* demonstrated that venetoclax increased the T cell infiltrate of MC38 colorectal cancers subcutaneously (*s.c*.) implanted in C57BL/6 mice (or that of CT26 mice implanted in BALB/c mice), sensitizing such tumors to PD-1 or PD-L1 blockade (30). We investigated whether such effects would also apply to orthotopic tumors, in particular MCA205 cutaneous fibrosarcomas and TC1 non-small cell lung cancers (NSCLC), which are not particular susceptible to *in vitro* killing by BCL2 inhibitors unless very high concentrations (≥10 μM) are used (Fig. S4A,B). Mice bearing orthotopic MCA205 fibrosarcomas (under the skin) or TC1 NSCLC (in the lung) were treated with intraperitoneal (*i.p.*) injection of navitoclax or venetoclax, followed by high-dimensional immune profiling of their T cells (Fig. S4C). PD-1 and CTLA-4 expression by CD8^+^ cytotoxic T lymphocytes (CTL) and CD4^+^Foxp3^+^ regulatory T cells (Treg) were consistently upregulated by navitoclax or venetoclax in TC1 (Fig. S4D-G) or MCA205 (Fig. S4H-I) bearing mice. Consistently, navitoclax and venetoclax sensitized MCA205 or TC1 tumors to PD-1 blockade, hence accentuating tumor growth reduction that can be conveniently monitored by an intrathoracic luciferase-dependent chemoluminescence signal (for TC1, Fig. S5A-E) and measuring the size of *s.c*. tumors (for MCA205, Fig. S5F-I).

**Figure 3:**
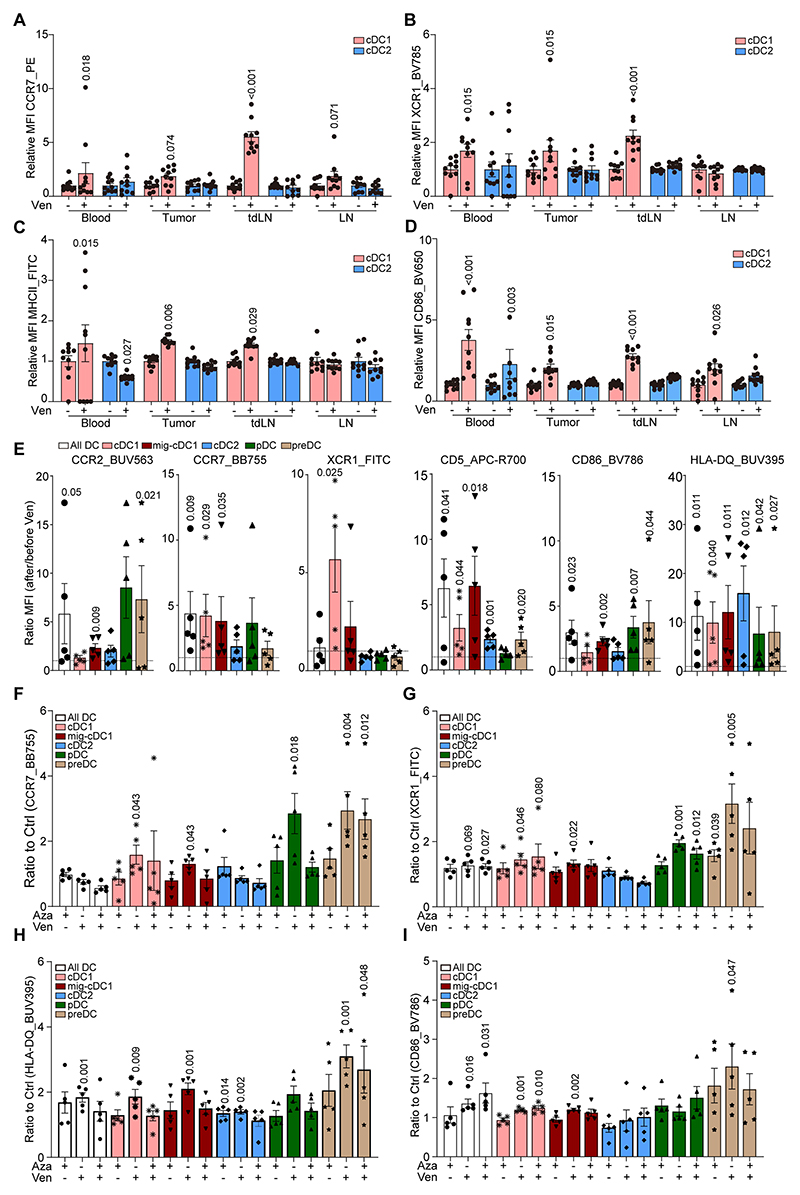
Pharmacological inhibitors of BCL2 activate conventional Type 1 DCs *in vivo*. (**A-D**) Orthotopic MCA205 fibrosarcoma-bearing mice received two intraperitoneal (*i.p.*) injections venetoclax or solvent controls (Ven) at day 0 (when tumors became palpable) and day 2. The blood, tumor, tumor-draining lymph node (tdLN), and non-tumor-draining LN (inguinal LN on the opposite side of the tumor) were harvested at day 4 and dissociated into single cell suspensions for multiplex immunostaining and flow cytometric analysis. The mean fluorescence intensity (MFI) of indicated markers on the Type 1 conventional DCs (cDC1, defined as F4/80^-^ MHC-II^+^CD11c^+^CD103^+^CD11b^-^ among viable leukocytes) as well as cDC2 cells (CD103^-^CD11b^+^) were normalized to the average value of the Sol condition, which are depicted as scattered dot plots (n=10 animals/group). Statistical significance was calculated using one-way ANOVA test with Dunnett’s multiple comparisons, as compared to Sol. (**E**) Cryopreserved PBMCs from acute myeloid leukemia (AML) patients, before- and after- (1 week) treatment with Ven plus azacytidine (Aza), were subjected to multiplex immunostaining and high dimensional flow cytometric analysis to quantify the expression of distinct DC activation related markers on the surface of different subpopulations. The ratio of MFI (after : before treatment) of CCR2, CCR7, XCR1, CD5, CD86, and HLA-DQ on different DC subsets from individual patients are depicted as scatter plot (n=5 patients). (**F-I**) PBMCs from healthy donors were treated *in vitro* with Ven, Aza, or their combinations and were then subjected to the same staining and analyses as above. The ratio of MFI (mono or combined treatment: DMSO control) from individual healthy donors are depicted as scatter plots (n=5 donors). A paired t test was performed to calculate the p values.

Of note, navitoclax and venetoclax also upregulated the expression of the chemokine receptors CCR7 and XCR1, as well as the maturation marker MHC-II, to a variable degree on cDC1 cells (defined as CD103^+^CD11b^-^ cells within DCs, defined as the viable CD45^+^CD11c^+^F4/80^-^ MHC-II^hi^ population, Fig. S6A) in the blood, tumor infiltrate, and lymph nodes from mice bearing TC1 orthotopic lung cancers (Figure S6B-D), without any signs of a reduction in absolute numbers of such DCs in those organs (Fig. S6E-G). Most of these upregulations were exclusively identified on cDC1 cells but not the (CD103^-^CD11b^+^) cDC2 subpopulation. Similarly, the upregulation of CCR7, XCR1, MHC-II molecules and CD86 on cDC1 was observed upon BCL2 inhibition with venetoclax in the MCA205 tumor model (Fig. 3A-D). Venetoclax also induced other maturation markers including CD80 and CD83 on cDC1 (and cDC2 in some cases) cells from the blood, tumor and both the tumor-draining and non-draining lymph nodes of mice bearing orthotopic MCA205 fibrosarcomas (Fig. S6H,I). Importantly, venetoclax upregulated IL12p40 and CXCR3 (also known as CD183) specifically on cDC1 cells from tumors or tumor draining lymph nodes, Fig. S6J,K).

We validated these findings in five patients with acute myeloid leukemia (AML) that were treated with a combination of venetoclax and azacytidine. After one cycle of therapy (daily oral administration for one week), circulating cDC1s (defined as leukocytes lacking lymphocyte markers but expressing high levels of HLA-DR, with low levels of CD1c, CD14, CD88 and CD123, but high expression of CD141, Fig. S7A) expressed higher levels of CCR2, CCR7, XCR1, CD5, CD86 and HLA-DQ, as determined by high-dimensional immunofluorescence cytometry (Fig. 3E). These changes were specific to cDC1 (not cDC2) cells. In PBMCs from healthy donors, the treatment with venetoclax but not azacytidine induced the activation of these markers at the surface of cDC1 (Fig. 3F-I, Fig.S7B), suggesting that the *in vivo* effects on circulating cDC1s are indeed mediated by venetoclax. Of note, there was no sign of DC depletion in venetoclax-treated PBMCs (Fig. S7C).

Altogether, these results suggest that BCL2 inhibition activates cDC1s both in tumor-bearing mice and in human.

### Adoptively transferred BCL2 KO DCs improve immunosurveillance

Since iniDCs can be limitlessly expanded, it is possible to generate large batches of de-iniDCs for their adoptive transfer into mice (19, 31). De-iniDCs labelled with the fluorescent dye PKH26 were intravenously (*i.v.*) injected into orthotopic TC1 lung cancer-bearing mice. Such cells could be detected in the lymph nodes and lung tissue up to 72 hours post-injection.

**Figure 4:**
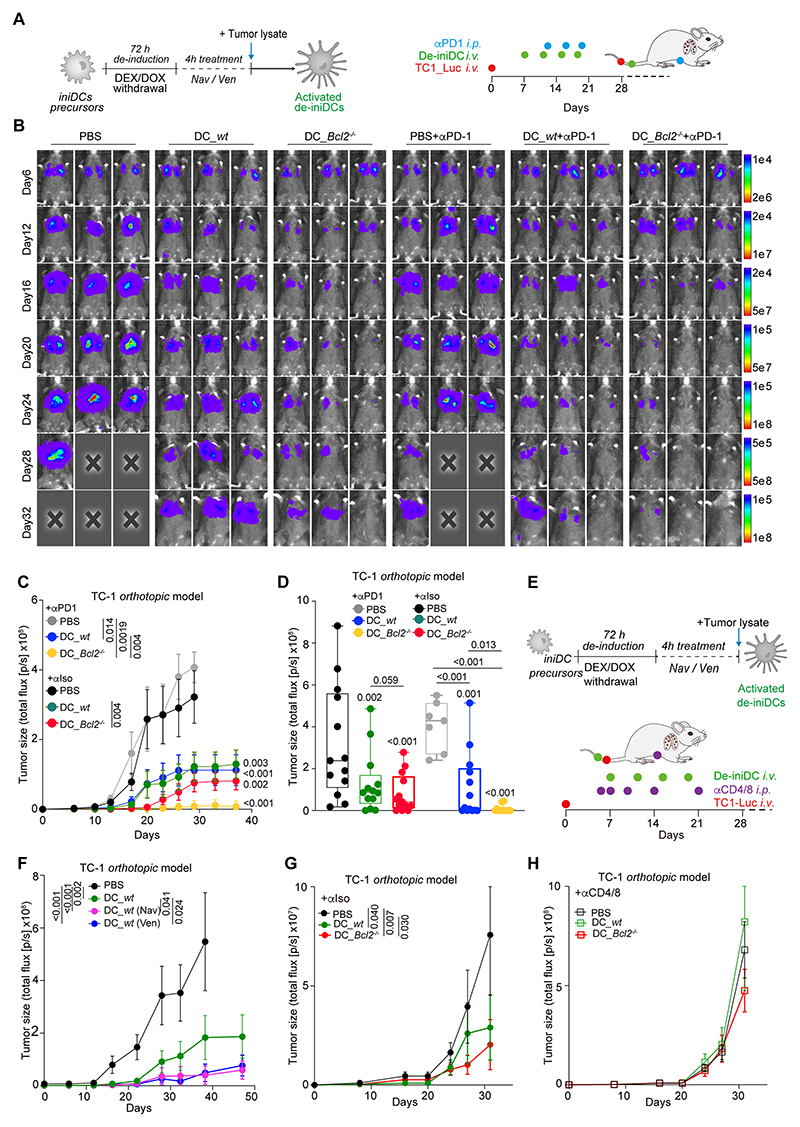
Bcl2 deletion or inhibition in DCs improves their potency in tumor control. (**A**) Schematic presentation of the experiment. Orthotopic lung cancers were established by intravenous (*i.v.*) injection of luciferase-expressing TC1 cells at day 0. Wild type (wt) or *Bcl2*^-/-^ de-iniDC_Cas9 cells were differentiated and fed with the lysate of TC1 cells to be activated before *i.v.* injection into the tumor-bearing mice. Once the tumors became detectable by bioluminescence (~ day 7), the animals were treated with 4 DC infusion cycles in combination with neutralizing antibodies to PD-1 (αPD-1) or corresponding isotype control antibody (αIso) as illustrated in the scheme. (**B**) Representative images of tumor development under different treatment conditions. Bioluminescence signals were quantified as total flux of photons to indicate tumor size in the lung and are reported as tumor growth curves as, means ± SEM (**C**). Final tumor size distribution at endpoint is shown in (**D**). (**E**) In some cases, de-iniDCs were pre-treated for 4 h with navitoclax (Nav) or venetoclax (Ven) before exposure to tumor lysate, and the adoptive DC transfer were used in combination with neutralizing antibodies to CD4 and CD8 (αCD4/CD8). (**F,G,H**) Tumor growth curves in mice receiving PBS as a control or de-iniDCs pretreated with navitoclax (Nav) or venetoclax (Ven) in vitro before injection (**F**) or de-iniDCs with different genotypes combined with control antibodies (**G**) or monoclonal antibodies depleting CD4^+^ and CD8^+^ T cells (**H**). Statistical significance was calculated by means of the Type II ANOVA test for tumor growth curves and one-way ANOVA test for the tumor size distributions, n = minimum of 7 mice/group. P-values are indicated to indicate statistical significance in intergroup comparisons.

Neither pretreatment of venetoclax nor *Bcl2* knockout did reduce the persistence of these cells *in vivo (*Fig S7D-F*)*. Both genetic and pharmacological inhibition of BCL2 enhanced the expression of MHC-II molecules on such cells (Fig S7G). Immunohistochemical detection of de-iniDCs (which are Cas9^+^) revealed their presence in TC1 lung cancers 72 hours after injection (Fig S7H). Importantly, *Bcl2*^-/-^ de-iniDCs were more abundant in the tumors than their WT counterparts (Fig S7I). Cytofluorimetric analyses of the lungs and lymph nodes of such mice revealed that the injection of *Bcl2*^-/-^ de-iniDCs or venetoclax-treated WT de-iniDCs (but not that of untreated WT-de-iniDCs) increased the local presence of CD8^+^ (including granzyme B^+^) T cells (Fig S8A,B). As compared to WT controls, intravenously injected *Bcl2*^-/-^ de-iniDCs were more efficient in controlling the growth of orthotopic TC1 NSCLC (Fig. 4A-D) and MCA205 fibrosarcomas (Fig. S8C-F), indicating that BCL2 inhibition in DCs alone is sufficient to improve immunosurveillance. WT de-iniDCs treated with navitoclax or venetoclax *in vitro* also became more efficient in controlling NSCLC upon their adoptive transfer than vehicle-treated WT de-iniDCs (Fig. 4E,F).

The tumor control mediated by *Bcl2*^-/-^ de-iniDCs was further enhanced by subsequent PD-1 blockade, as shown for both TC1 (Fig. 4A-D) and MCA205 cancers (Fig. S8C-F), but was lost upon depletion of T cells by means of neutralizing antibodies specific to CD4 and CD8 (Fig. 4G,H, Fig. S8G), as well as in nude mice lacking mature T cells due to the *Foxn1*^nu/nu^ mutation (Fig. S8H). Antibody-mediated blockade of IFNAR1 on venetoclax-treated DCs or *Bcl2*^-/-^ DCs reduced their capacity to control the growth of tumors and to extend animal survival (Fig. S8I,J). Finally, the growth control of M/D-driven mammary carcinomas by radiotherapy could be enhanced by venetoclax, and this improvement was abolished upon neutralization of IFNAR1 (Fig. S8K).

These results confirm that *Bcl2*^-/-^ DC exhibit a gain-of-function phenotype that improves T cell-mediated cancer immunosurveillance and that depends on Type 1 interferon signaling.

### BCL2 inhibitors stimulate immunosurveillance in a cDC1-dependent fashion

**Figure 5:**
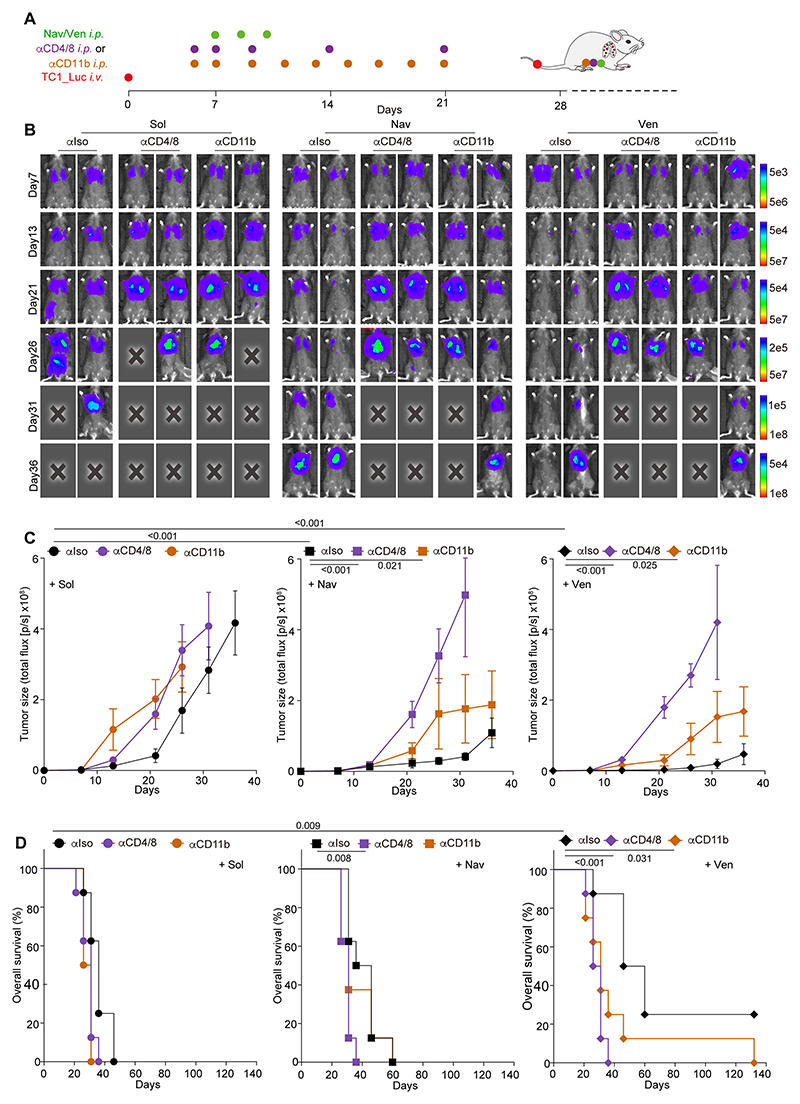
Systemic administration of Bcl2 inhibitors exerts immune-dependent anticancer efficacy. Orthotopic lung cancers were established by intravenous (*i.v.*) injection of luciferase-expressing TC1 cells on day 0. Once the tumors became detectable by bioluminescence (~ day 7), the animals were intraperitoneally (*i.p.*) treated with either solvent (Sol), navitoclax (Nav), or venetoclax (Ven) at day 7, 9, and 11 in combination with neutralizing antibodies to CD11b (αCD11b) or CD4 and CD8 (αCD4/CD8), or the isotype control antibody (αIso) as illustrated in the scheme (**A**). Representative images of tumor development under different treatment conditions are shown in (**B**). Bioluminescence signals were quantified as total flux of photons to indicate tumor sizes on the lung which are reported as tumor growth curves (**C**, mean ± SEM). The percentage of overall survival is reported in (**D**). Statistical significance was calculated by means of the type II ANOVA test (**C**), or logrank test (**D**), n = minimum of 8 mice/group. P-values are labelled in the figure and indicate statistical significance as compared with PBS or between the indicated groups.

To prove the importance of DCs for the anticancer effects of pharmacological BCL2 inhibitors, we used several strategies. First, we blocked the extravasation of myeloid cells (including DC) by means of a CD11b-blocking antibody (32, 33) and found that this manipulation depleted the cDC2 population in blood, tumors and tumor-draining lymph nodes (Fig. S9A), but did not change the number of cDC1 cells (which are CD11b^-^ as illustrated in the gating strategy in Fig. S6) (16) in the blood, but prevented cDC1 cells and in particular migratory XCR1^+^ cDC1 cells from entering the tumor bed and (partially) the tumor-draining lymph nodes (Fig. S9B,C). In addition, CD11b blockade as well as T cell depletion compromised the tumor growth-reducing effects (Fig. 5 A-C) and animal survival-enhancing of navitoclax and venetoclax against TC1 lung cancers (Fig. 5 D). This finding could be recapitulated for MCA205 fibrosarcomas, which failed to respond to BCL-2 inhibitors upon blockade of CD11b, antibody-mediated depletion of T lymphocytes or in the context of genetically determined athymia (Fig. S9D-H). Thus, not only T cells but also myeloid cells are required for pharmacological BCL2 inhibitors to reduce tumor growth.

**Figure 6:**
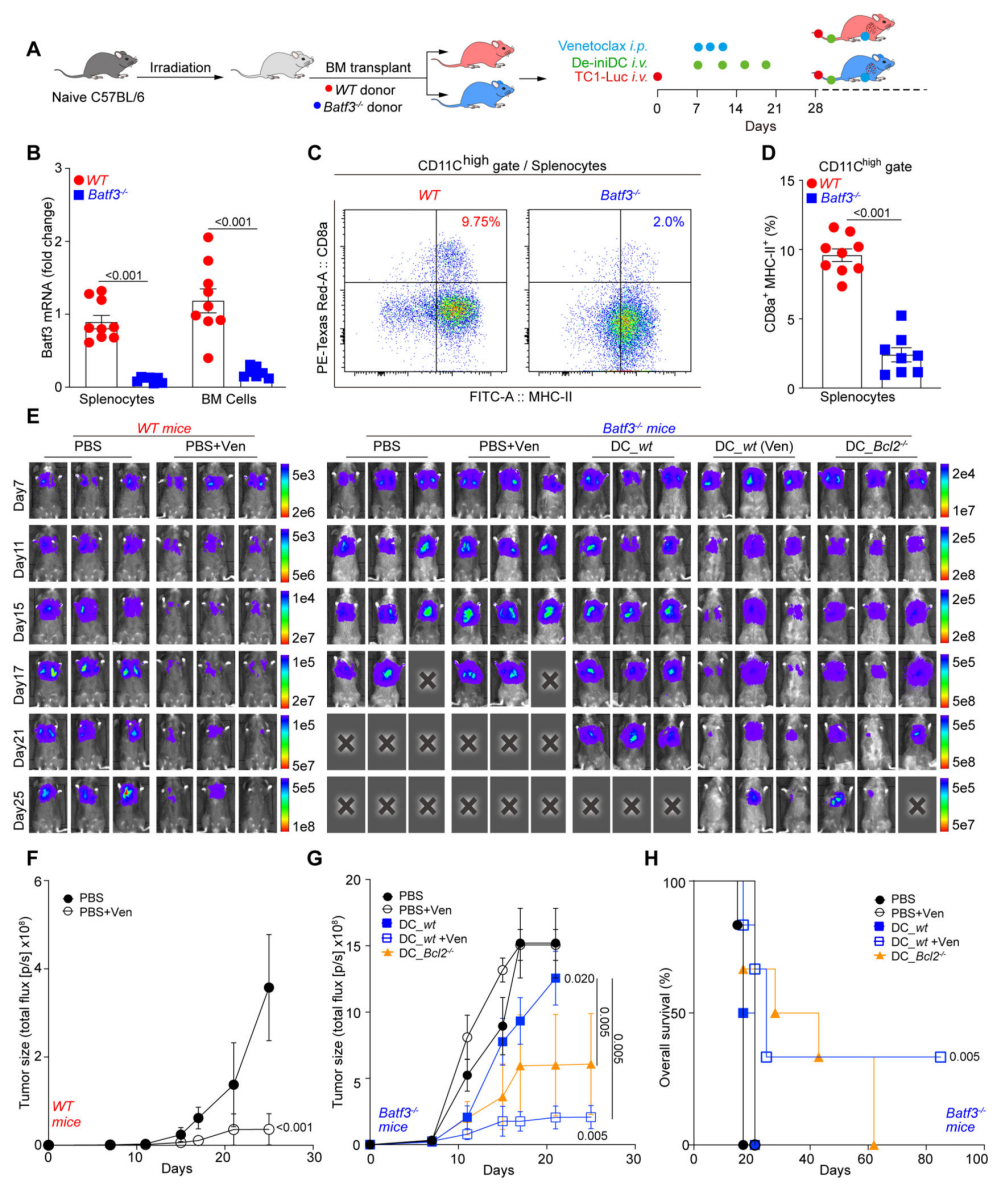
Genetical depletion of Type 1 conventional DCs interferes the antitumor effect by Bcl2 inhibitors. **(A)** Lethal dose irradiated mice were transplanted with bone marrow from WT or Batf3-KO donors to establish a conditional change in the immune system. In a pilot experiment, the splenocytes and BM cells were isolated from bone marrow reconstituted mice to verify the deletion of *Batf3* by qPCR (**B**) and the depletion of cDC1s by flow cytometric analysis (**C,D**). For the tumor growth experiment, orthotopic lung cancers were established in bone marrow reconstituted mice by *intravenous* (*i.v.*) injection of luciferase-expressing TC1 cells on day 0. Wild type (wt) or *Bcl2*^-/-^ de-iniDC_Cas9 cells were differentiated and activated with the lysate of TC1 cells before *i.v.* injection into the tumor-bearing mice. Once the tumors became detectable by bioluminescence (~ day 7), the animals were infused with 4 cycles of de-iniDCs, with or without combination of *intraperitoneal* (*i.p.*) treatment by either solvent (Sol) or venetoclax (Ven). Representative images of tumor development under different treatment conditions are shown in (**E**). Bioluminescence signals were quantified total flux to indicate tumor size on the lung which are reported as tumor growth curves (**F,G**, mean ± SEM) The percentage of overall survival is reported in (**H**). Statistical significance was calculated by means of the type II ANOVA test (**F,G**), or logrank test (**H**), n = minimum of 6 mice/group. P-values are labelled in the figure and indicate statistical significance as compared with PBS + Sol or between the indicated groups.

Next, we determined which DC subset is involved in the response to BCL2 inhibitors. *Batf3*^-/-^ mice lack cDC1 cells (13), and cancers evolving in such mice fail to respond to PD-1 blockade (14, 15) or other types of immunotherapies (16–18, 34, 35). We reconstituted lethally irradiated WT C57BL/6 mice with the bone marrow from syngeneic WT controls or *Batf3*^-/-^ mice (Fig. 6A) and confirmed that infusion of *Batf3*^-/-^ bone marrow cells caused *Batf3*^-/-^ deficiency in the spleen and bone marrow (Fig. 6B) with the consequent reduction in cDC1 cells (defined as CD11c^high^CD8α^+^MHC-II^+^, Fig S10A) in lymphatic organs (Fig. 6C,D). Venetoclax injections controlled orthotopic TC1 lung cancers in WT bone marrow-reconstituted mice, but completely failed to do so in mice that were reconstituted with bone marrow from *Batf3*-deficient donors (Fig. 6E-H). However, TC1 tumors established in mice with a *Batf3*-deficient immune system responded to venetoclax upon the adoptive transfer of WT de-iniDCs. Moreover, such tumors evolving in *Batf3*-deficient hosts could be partially controlled by the infusion of *Bcl2*^-/-^ de-iniDCs (Fig. 6E-H).

**Figure 7:**
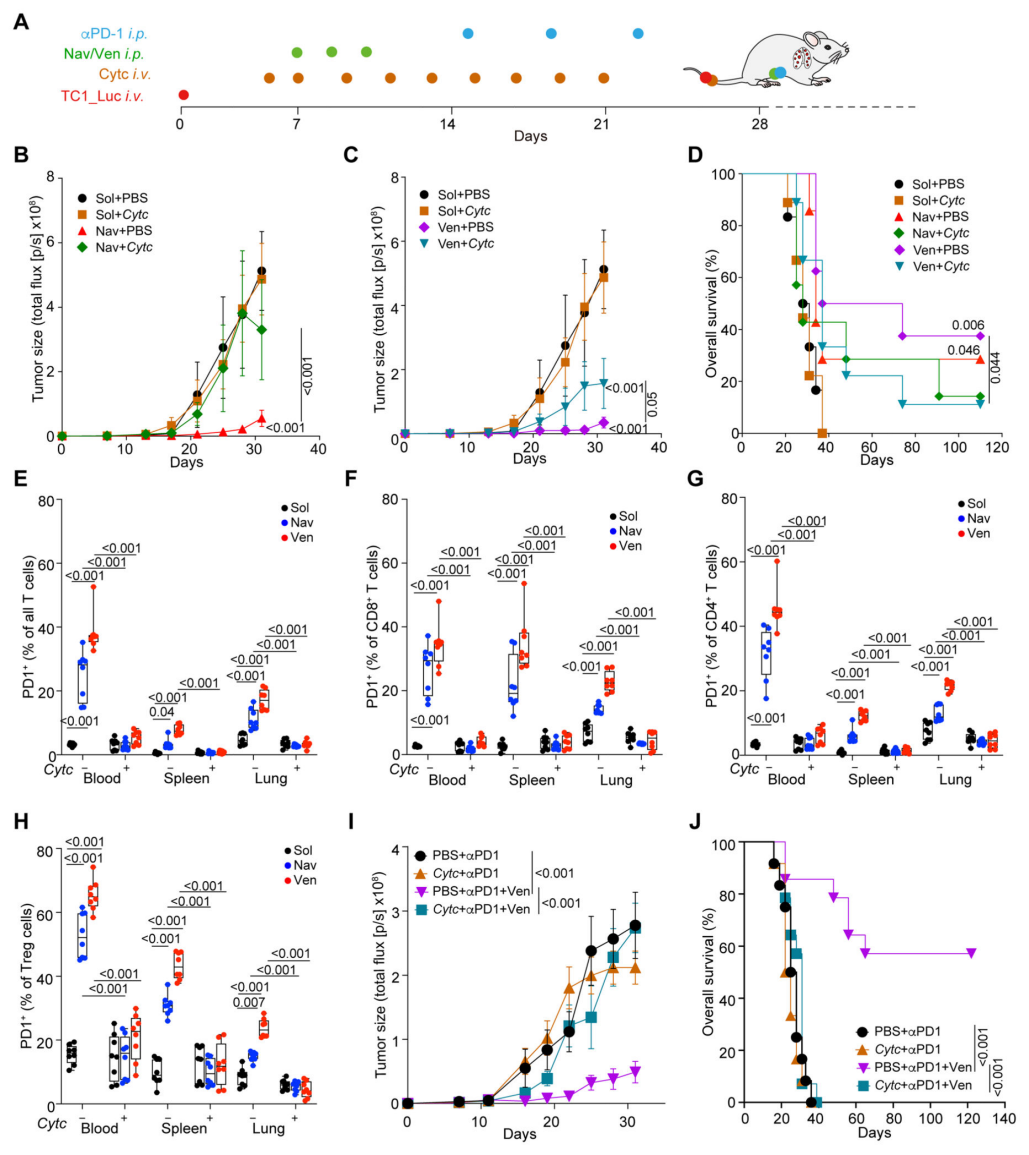
Systemic depletion of Type 1 conventional DCs interferes the antitumor effect by Bcl2 inhibitors. Orthotopic lung cancers were established in wild type C57BL/6 mice by *intravenous* (*i.v.*) injection of luciferase-expressing TC1 cells on day 0. Once the tumors became detectable by bioluminescence (~ day 7), the animals were subjected to treatment as schemed in (**A**). Briefly, mice were pre-treated (*i.v.*) with either 5 mg/mouse of cytochrome C (*Cytc*) or the corresponding vehicle (PBS) before other treatment and maintained the injection every other day for two weeks. Then intraperitoneal (*i.p.*) treatment with either solvent (Sol), navitoclax (Nav), or venetoclax (Ven) was applied, and the combination with PD-1 (αPD-1) blockade or equivalent isotype antibody (αIso) followed 8 days after chemical treatment. Bioluminescence signals were quantified as total flux to indicate tumor size on the lung which are reported as tumor growth curves (**B**,**C**, mean ± SEM). The percentage of overall survival is reported in (**D**). Statistical significance was calculated by means of the type II ANOVA test (**B,C**), or logrank test (**D**), n = 9 mice/group. (**E-H**) Orthotopic TC1 lung tumor-bearing mice were treated as described above. Spleen, lung, and blood were harvested 7 days later and were dissociated into single cell suspensions for multiplex immunostaining and flow cytometric analysis. The percentage of PD-1^+^ cells in total T cells (**E**), CD8^+^ T cells (**F**), CD4^+^ T cells (**G**), as well as Tregs (defined as CD4^+^FOXP3^+^CD25^+^,**H**) among total T cells were evaluated with FlowJo and depicted as dot plots (n=8 animals/group). Statistical significance was calculated using one-way ANOVA test. (**I,J**) Lung tumor growth in TC-1 lung cancer-bearing mice that were treated with *Cytc*, Ven, antiPD-1, or their combinations, were monitored and depicted as growth curves (**I**); overall survival is reported as Kaplan–Meier curves (**J**). Statistical analysis was performed as (**B-D**). P-values are labelled in the figure to indicate statistical significance as compared with PBS + Sol or between the indicated groups.

The aforementioned results suggest that Batf3-dependent cDC1 cells are required for the anticancer effects of BCL2 inhibitors. To confirm the involvement of cDC1 cells in the response to navitoclax and venetoclax, we resorted to another method of cDC1 depletion consisting in the repeated intravenous injection of Cyt *c* (21). We confirmed that *i.v.* injection of Cyt *c* (5 mg/mouse every other day) caused the selective depletion of cDC1 (including that of XCR1^+^ migratory cDC1) but no cDC2 cells in the blood, tumor, and tumor-draining lymph nodes from mice bearing MCA205 fibrosarcomas (Fig. S9A-C) or TC-1 lung cancers (Fig S10B-D). Importantly, Cyt *c* injections reduced the antitumor effects of navitoclax and venetoclax against TC1 lung cancers (Fig. 7A-D), as well as against MC205 fibrosarcomas (Fig. S10E-G). Depletion of cDC1 cells also abolished the BCL2 inhibitor-induced upregulation of PD-1 and CTLA-4 on CD8^+^ CTLs and CD4^+^ T cells including Foxp3^+^ Tregs (Fig.7 E-H, Fig. S10H-K). Accordingly, the synergistic interaction between venetoclax and PD-1 blockade with respect to tumor growth reduction and animal survival was lost when cDC1 were depleted by repeated Cyt *c* injections (Fig.7 I,J).

Altogether, these results indicate that BCL2 inhibitors require cDC1 cells to mediate their anticancer effects, and the cooperation with immune checkpoint inhibitors.

## Discussion

Most anticancer drugs have been designed to selectively kill malignant cells (efficacy) and to spare normal cells (toxicity). However, it has turned out that antineoplastics that are clinically efficient often stimulate an anticancer immune response that accounts for the long-term effects of the medication beyond therapy discontinuation (36). Prominent examples for this mode of action include anthracyclines, oxaliplatin and taxanes that induce immunogenic cell death (ICD) of malignant cells, hence stimulating an immune response against dead-cell antigens that is mediated in the first place by dendritic cells (that have to engulf portions of stressed and dying cancer cells) which subsequently present tumor-associated antigens to T cells recruited into the tumor microenvironment (37). However, in a certain sense, such ICD-inducing cytotoxicants act “on-target” because they primarily stress and kill malignant cells. Many other anticancer drugs appear to have additional “off-target” effects in the sense that they directly affect different immunostimulatory or immunosuppressive circuitries as this has been well documented for imatinib (which activates the DC-NK cell dialogue) (37) or 5-fluorouracil that depletes myeloid derived suppressor cells from the tumor bed (38). Our present work suggests that BCL2 inhibitors may also mediate therapy-relevant off-target effects on the immune system, in particular on DCs.

There are several lines of evidence that plead in favor of indirect, immune-dependent effects of BCL2 inhibition by the clinically approved drug venetoclax, as well as by the experimental inhibitor navitoclax. First, MCA205 and TC1 cancers largely failed to respond to treatment with navitoclax and venetoclax when different immune effectors were deficient, as shown for constitutively athymic mice, depletion of T cells in adult mice, the knockout of Batf3 (causing constitutive absence of cDC1 cells), as well as for the depletion of cDC1 cells by Cyt *c* injections. Second, echoing a prior report dealing with ectopic (*s.c*.) MC38 colorectal cancers (30), orthotopic TC1 lung cancer and cutaneous MCA205 fibrosarcomas implanted in syngeneic hosts responded to PD-1 blockade more vigorously upon pre-treatment with navitoclax and venetoclax. Hence, systemic BCL2 inhibition cannot reduce the growth of solid tumors (such as MCA205, MC38 and TC1) via direct antineoplastic effects but relies on a cellular immune response.

Previous reports indicate that the BCL2 inhibitor venetoclax causes a depletion of various (B, T and NK) circulating lymphocyte subsets after one year of treatment of patients with chronic lymphoid leukemia (39). This contrasts with another report showing that short-term treatment of healthy volunteers increases the fraction of CD4^+^ and CD8^+^ effector memory cells (TEM and TEMRA) but decreases the proportion of non-effector cells (TN and TCM), in accord with findings obtained by *in vitro* treatment of circulating leukocytes (30). Moreover, venetoclax fails to impair the proliferation of activated primary human T cells *in vitro* (40) and rather directly activates T cells, increasing their cytotoxic potential against acute myeloid leukemia cells *in vitro* and *in vivo* through stimulatory effects on mitochondrial generation of reactive oxygen species (41). Hence, the available evidence from human studies suggests that venetoclax does not compromise immune function and rather stimulates anti-leukemic T cell responses. However, no information on the prognostic or predictive value of such T cells have been reported for venetoclax-treated patients. As shown here, venetoclax activates cDC1 cells in humans both *in vitro*, in cultured blood leukocytes from healthy donors, and *in vivo*, in patients with acute myeloid leukemia, in whom circulating cDC1 cells exhibited the upregulation of several markers of DC activation.

Prior studies have demonstrated the importance of cDC1 cells for the outcome of immunotherapies and targeted therapies (42–45). In view of a possible survival deficit of BCL2-inhibited DCs (46–50), we carefully examined the possibility that BCL2 inhibition would deplete such cells. However, venetoclax treatment of tumor-bearing mice or AML patients failed to deplete DCs, and *Bcl2*^-/-^ de-ini-DCs that were transferred into NSCLC-bearing mice infiltrated tumors more efficiently than their WT counterparts. In addition, it appears that BCL2 inhibition improves the function of de-iniDCs (in vitro) and cDC1 cells (in vivo). This was coupled to multiple signs of DC activation, with an increase in the expression of chemokine receptors, cytokines, co-stimulatory receptors and, perhaps most importantly, a marked stimulation of a Type-1 interferon response that is likely due to the activation of the mtDNA/cGAS/STING/IRF3 pathway. Indeed, knockout or blockade of IFNAR1 was sufficient to block the enhancement of antigen presentation by BCL2 inhibition in de-iniDCs in vitro, as well as their tumor growth controlling activity in vivo.

At the preclinical level, our study provides strong evidence that BCL2 inhibitors act on-target on DCs (and in particular the cDC1 subset) to stimulate an anticancer immune response. First, pharmacological inhibition or genetic inhibition of BCL2 in de-iniDCs (which transcriptionally and functionally resemble cDC1 cells and can functionally reconstitute cDC1-deficient *Batf3*^-/-^ mice with respect to cancer immunosurveillance) enhances their antigen-presenting function, induces a marked type-1 interferon response and upregulates DC activation markers *in vitro*. Second, venetoclax and navitoclax cannot enhance the function of BCL2 KO de-ini-DCs any further, strongly suggesting that they act on BCL2 rather than on other proteins from the BCL2 family. Accordingly, BCLXL reportedly improves cDC1 function if overexpressed, not if inhibited, in another experimental system (51). Third, DC (and specifically cDC1) activation is also observed in mice or humans treated with BCL2 inhibitors in vivo. Fourth, adoptive transfer experiments revealed that BCL2-deficient de-iniDCs are superior to WT de-iniDCs in improving T cell-dependent immunosurveillance in mice. This superior effect was obtained upon *i.v.* injection of genetically engineered DCs into tumor bearing wild type mice, as well as into *Batf3*^-/-^ recipients. Fifth, when WT de-iniDCs are treated *ex vivo* with navitoclax or venetoclax and subsequently injected into tumor bearing mice, they mediate enhanced cancer control. Sixth, constitutive absence of cDC1 cells (due to the *Batf3* knockout) or their acquired deficiency (due to injection of Cyt *c*) reduced or abolished the therapeutic effects of navitoclax or venetoclax against established solid tumors. Finally, the depletion of cDC1 cells prevented the BCL2 inhibitor-induced upregulation of CTLA-4 and PD-1 on T lymphocytes, suggesting that these T cell effects are secondary to DC stimulation.

In sum, the available evidence suggests that (one of) the primary target(s) of BCL2 inhibitors are DCs from the cDC1 subset. BCL2 appears to act as an endogenous checkpoint of DC function, hence exemplifying a druggable target that normally restrains DC function. If BCL2 inhibitors constitute a novel class of immune checkpoint inhibitors acting on DCs they might have broad anticancer effects (especially if combined with “classical” immunotherapies targeting PD-1 or PD-L1) that transcend their direct effects on malignant cells. Most clinical trials involving venetoclax are currently targeting hematopoietic cancers, often in combination with other drugs conceived for their cell-autonomous antineoplastic action. However, a few trials involve combinations with PD-1 or PD-L1 blockade (NCT03390296, NCT03969446, NCT04277442, NCT05388006 listed in https://clinicaltrials.gov) for the treatment of lymphomas or leukemias. Moreover, one Phase I study is evaluating the safety of venetoclax plus pembrolizumab in the treatment of PD-L1^high^ NSCLC (NCT04274907). In this context, it will be important to monitor the immunostimulatory effects of BCL2 inhibitors on circulating and tumor-infiltrating cDC1 cells.

## Materials and Methods

### Cell Culture and related reagents

RPMI-1640 medium (Cat# 61870010), DMEM medium (Cat# 10566016), HEPES (CAT# 15630056), sodium pyruvate (Cat# 11360070), phosphate-buffered saline (PBS, Cat# 20012027), penicillin-streptomycin (Pen/Strep, 10,000 U/mL, Cat# 15140122), and TrypLE™ Express (Cat# 12604013) were purchased from Life Technologies (Carlsbad, CA, USA). Fetal bovine serum (FBS, Cat# F7524), β-mercaptoethanol (Cat# M3148), dexamethasone (Cat# D0700000), doxycycline hyclate (Cat# D3000000), and PKH26 staining kit (Cat# PKH26GL-1KT) were purchased from Sigma (St. Louis, MO, USA). Recombinant murine GM-CSF (Cat# 315-03) was obtained from Peprotech (Cranbury, NJ, USA). Unless otherwise indicated, all plasticware was purchased from Corning. Life Sciences (Corning, NY, USA).

The parental iniDCs were kindly shared by Cornelia Richter and colleagues (20), iniDCs stably expressing CRISPR Cas9 (iniDC_Cas9) were established by transduction with Edit-R lentiviral CAG-Blast-Cas9 nuclease particles (Cat# VCAS10129, Horizon Discovery, Waterbeach, UK) followed by cloning and validation as previously published (19). All other gene-edited iniDC cell lines were generated by transfecting iniDC_Cas9 cells with specific crRNA + tracrRNA, followed by single cell sorting and immunoblotting for knockout validation. As basic DC medium RPMI-1640 with 10% decomplemented FBS, 1 mM sodium pyruvate, 10 mM HEPES, and 1x Pen/Strep was used. β-mercaptoethanol (at a final concentration of 50 μM) and recombinant GM-CSF (at a final concentration of 10 ng/mL) was freshly added. IniDCs and derivative cell lines are immortalized under the induction of Dex/Dox (Dex at 100 nM + Dox at 2 μM). Dex/Dox removal (“deinduction”) led to a halt in proliferation and differentiation into immature DCs (“de-iniDCs”) that were used for experiments. The B3Z hybridoma T cells were kindly provided by Sebastian Amigorena and maintained with DC medium supplemented with β-mercaptoethanol (50 μM). MCA205 fibrosarcoma (Cat# SCC173, Sigma-Aldrich) and TC1 non-small cell lung cancer cells expressing luciferase (TC1_Luc, kindly shared by T.-C. Wu (52)) were cultured with DMEM medium containing 10% decomplemented FBS and 1x Pen/Strep. All cell lines were regularly checked for contamination with the MycoStrip™ - Mycoplasma Detection Kit (Cat# rep-mysnc-100, InvivoGen, Toulouse, France). The cell lines were maintained in culture for a maximum of 15 passages from thawing, and fresh frozen stocks were prepared with cells in the 2^nd^ or 3^rd^ passages and confirmed mycoplasma-free before freezing.

### Chemicals and antibodies

The Bcl2 family inhibitors including ABT-199/venetoclax (Cat# HY-15531), ABT-263/navitoclax (Cat# HY-10087), ABT-737 (Cat# HY-50907), A-1331852 (Cat# HY-19741), S63845 (Cat# HY-100741), and WEHI-539 (Cat# HY-15607A) were purchased from MedChemExpress (Monmouth Junction, NJ, USA). Lipopolysaccharides (LPS, Cat# L2654) albumin from chicken egg white (OVA, Cat# A5503), and Cytochrome c from equine heart (Cat# C7752) were obtained from Sigma. Antibodies employed for immunoblot and immunofluorescence such as αβ-actin (HRP, Cat# ab49900), αBCL2 (Cat# ab182858), and αLC3B (Cat# ab192890) were from Abcam (Cambridge, UK); αdsDNA marker (Cat# sc-58749) were purchased from Santa Cruz Biotechnology (Dallas, TX, USA); αATG7 (Cat# 8558), αBAX (Cat# 2772), and αcleaved caspase3 (Cat# 9661), αCas9 (Cat# 19526), and the mouse-reactive STING pathway antibody sampler kit (Cat# 16029) were from Cell Signaling Technology (Danvers, MA, USA). *In vivo* neutralizing antibodies to PD-1 (Cat# BE0273), CD4 (Cat# BE0003-1), CD8 (Cat# BE0061), CD11b (Cat# BE0007), IFNAR (Cat# BE0241) and corresponding isotype controls (Cat# BE0090, BE0083) were purchased from BioXcell (Lebanon, NH, USA). *In vitro* neutralizing antibodies to mouse CD70 (Cat# 104603), CD80 (Cat# 1047481), CD86 (Cat# 159302), IL12 (Cat# 505308), and corresponding isotype controls (Cat# 400502, 400940) were obtained from Biolegend (San Diego, CA, USA). Antibodies for ELISA, including αIL1β (Cat# 503502), biotin-conjugated αIL1β (Cat# 515801), αIL2 (Cat# 503702), biotin-conjugated αIL2 (Cat# 503804), IL6 (Cat# 504502), and biotin-conjugated αIL6 (Cat# 504602) came from Biolegend. Mouse monoclonal antibodies employed for high dimensional flow cytometry: αCCR2 BV650 (Cat# 150613), αCCR7 Alexa Fluor 488 (Cat# 120110), αCCR7 PE (Cat# 120106), αCD3 APC (Cat# 100236), αCD11c APC (Cat# 117310), αCD16/32 (Cat# 101302), αCD25 BV650 (Cat# 100236), αCD45 Alexa Fluor 700 (Cat# 103116), αCD80 PE (Cat# 104708), αCD80 PercP-Cy5.5 (Cat# 104722), αCD86 BV650 (Cat# 105036), αCTLA-4 PE-Cy7 (Cat# 106314), αF4/80_BV785 (Cat# 123141), αIL12p40_APC (Cat# 505206), αMHC-II BV650 (Cat# 107641), αMHC-II FITC (Cat# 115006), and αXCR1 BV785 (Cat# 148225) were from Biolegend; αCD4 eFluor450 (Cat# 48-0042-82), αCD8a PercP-Cy5.5 (Cat# 45-008182), αCD11b eFluor450 (Cat# 48-0112-82), αCD11b BUV395 (Cat# 363-0112-82), αCD11c APC-eFluor780 (Cat# 47-0114-82), αCD40 eFluor450 (Cat# 48-0402-82), αCD45 APC-eFluor780 (Cat# 103116), αCD69 E-Cy5 (Cat# 15-0691-82), αCD83 PE-Cy7 (Cat# 25-083942), αF4/80_Alexa Fluor 700 (Cat# 56-4801-82), αFOXP3 FITC (Cat# 11-5773-82), αPD-1 PE (Cat# 12-9985-82), αPD-L1 PE(Cat# 12-5982-82), αMHC-II PE (Cat# 12-5321-82), αOVA257-264 (SIINFEKL) peptide bound to H-2Kb APC (Cat# 17-5743-82) came from eBioscience/Life Technologies; αCD183 BV650 (Cat# 740630) and αCD103 BUV661 (Cat# 750718), were purchased from BD. Flow cytometry related monoclonal antibodies for human samples, αCD3 BV650 (Cat# 300468), αCD8 APC/Fire810 (Cat# 344764), αCD11b BV570 (Cat# 101233), αCD14 SparkB550 (Cat# 367148), αCD16 BV650 (Cat# 302042), αCD19 BV650 (Cat# 302238), αCD20 BV650 (Cat# 302336), αCD40 PE/Cy7 (Cat# 334322), αCD45 PercP (Cat# 368506), αCD45RA Spark NIR685 (Cat#304 168), αCD80 PE (Cat# 305208), αCD88 APC/Fire750 (Cat# 344316), αCD123 PE/Daz594 (Cat# 306034), αCD141 BV421 (Cat# 344114), αCX3CR1 BV711 (Cat# 341630), αHLA-DR PE/Fire810 (Cat# 307683), and αXCR1 FITC (Cat# 372612) were purchased from Biolegend; αCD1c SB436 (Cat# 62-0015-42), αCD206 PP/eF710 (Cat# 46-2069-41), αCD69 PE/Cy5.5 (Cat# MHCD6918), αCD274 (PD-L1) PE/Cy5 (Cat# 15-5983-42), and αchickIgY-AF647 (Cat# A21449) were purchased from Life Technologies, α CD5 APC/R700 (Cat# 565121), α CD11c BUV805 (Cat# 742005), α CD25 BV605 (Cat# 562660), α CD38 BUV615 (Cat# 751138), α CD83 BV510 (Cat# 563223), α CD86 BV786 (Cat# 740990), α CD169 BUV661 (Cat# 750363), α CD178 APC (Cat# 564262), α CD192(CCR2) BUV563 (Cat# 749076), α CD197(CCR7) BB755 (Cat# 624391), α CD279 (PD1) BV750 (Cat# 747446), and α HLA-DQ BUV395 (Cat# 742614) were purchased from BD; αCD4 Cfl.YG584 (Cat# R7-20041) was purchased from CyTek (Fremont, CA, USA); α CADM1 (Cat# CM004-3) was purchased from MBL international (Woburn, MA, USA); α SLAN VioBlue (Cat# 130-119-868) was purchased from Miltenyi Biotec (Bergisch Gladbach, Germany). The Live/dead Yellow Fixable Dye (Cat# L34959), Hoechst 33342 (Cat# H3570), Alexa Fluor® conjugated 2^nd^ antibody, and MitoTracker™ Orange CMTMRos (Cat# M7510) were from Life Technologies, and ViaDye™ Red Fixable Viability Dye (Cat# R7-60008) came from CyTek.

### Genome-wide CRISPR KO screening

The Mouse Brie CRISPR knockout pooled library was a gift from David Root and John Doench (53). The library was obtained as lentiviral particles from Addgene (Cat#73633). For transduction, iniDC_Cas9 cells were expanded and seeded at 3x10^6^ cells/ well in tissue culture-treated 12-well plates with complete DC medium containing β-mercaptoethanol and GM-CSF. The transduction mix was prepared by mixing 5 μg polybrene with 200 μl lentiviral particles and 800 μl DC medium for each well, corresponding to a multiplicity of infection (MOI) of 0.3. The transduction mix was added dropwise into the media and was well mixed by gentle pipetting. Plates were centrifuged at 650 g for 2 h at room temperature, then transferred incubated overnight at 37°C and 5% CO_2_. An additional control well was prepared with the same procedure without the lentiviral particles. The next day, infected cells were detached by trypsin and transferred to T175 tissue culture flasks at a density of 2x10^7^ cells/flask in DC medium containing β-mercaptoethanol, GM-CSF, and Dex/Dox. The control well was transferred into a T25 flask. Forty-eight hours later, all transfected cells as well as the control cells were treated with 10 μg/mL puromycin in the same medium, which was maintained for 4 days until all cells in the control flask were killed. Supernatant in the T175 flasks containing transduced cells replaced with fresh DC medium containing β-mercaptoethanol, GM-CSF, and puromycin, but no Dex/Dox. After 4 days of differentiation, the cells were incubated with soluble OVA (2 mg/mL in DC medium) for 18 h and processed for immunostaining. Briefly, cells were collected by scrapping and washed once with PBS. Final cell density was adjusted to 2.5x10^7^ cells/mL in FACS buffer (1% BSA in PBS). An aliquot of untreated control cells was spun down, resuspended in 1 mL FBS containing 10% DMSO, and cryopreserved at -80°C. The cellular suspension was divided into aliquots of 5x10^7^ cells in FACS tubs, which were firstly incubated with 10 μg Fc blocking antibody (anti-CD16/32) for 10 min, then centrifuged at 400 g for 5 min and resuspended in 2 ml FACS buffer containing 10 μg of anti-mouse CD80 PE and 5 μg anti-mouse SIINFEKL bound to H-2Kb APC antibodies. Cells were stained at 4°C in the dark, for 30 min, then centrifuged at 400 g for 5 min, and finally resuspended with 2.5 mL FACS buffer for continuous cell sorting on a BD FACSAria™ Fusion flow cytometer (BD, Franklin Lakes, NJ, USA). Just before sorting, 1 μg/mL DAPI was added to the stained cells for viability assessment. The CD80^high^SIINFEKL^high^ DAPI^-^ cells were collected, and cryopreserved at - 80°C. The same procedure was repeated to get biological replicates. Genomic DNA was extracted by using the Blood & Cell Culture DNA Midi Kit (Cat# 13343) from Qiagen (Hilden, Germany) following the manufacture’s protocol.

PCR of sgRNAs for Illumina sequencing was performed using the NEBNext Ultra II Q5 Master Mix (Cat# M0544X, New England BioLabs Ipswich, MA, USA), with the P5&P7 primers (sequence can be found in the Addgene (Cat#73633) data sheet) synthesized by Eurofins Genomics (Ebersberg, Germany). PCR reaction system was optimized as 50 μL/reaction containing 1 μM of P5 primer mix, 1 μM of P7 primer with distinct barcode sequence, 500 ng genomic DNA, and 25 μL of the PCR master mix. PCR parameters were adapted from the NEBNext® Ultra™ II DNA Library Prep Kit for Illumina® (NEB #E7645S/L, #E7103S/L), with an annealing temperature of 72°C and 26 denaturation-annealing-extension cycles. PCR products were purified using the QIAquick PCR & Gel Cleanup Kit (Cat# 28506, Qiagen) strictly following the manufacture’s protocol. The quality control of purified PCR products was verified on an Agilent 2100 Bioanalyzer with the High Sensitivity DNA Kit (Agilent, Santa Clara, CA, USA). Next generation sequencing was performed on an Illumina NextSeq 550 System using single reads, with 20% PhiX to improve library diversity, and covered >250 reads per sgRNA. The obtained fastq files were processed by using the MAGeCK package (22, 54). The <*mageck count*> command was used to generate per-sgRNA read count table by matching single-end reads with sgRNA sequences provided in the brie library data sheet that can be downloaded from Addgene (Cat#73633) (53). The <*mageck test*> subcommand was used to perform MAGeCK RRA (22, 54) for the comparison between the sorted and unsorted condition and generated a *gene_summary_txt* file containing the statistical contents for the identification of hits.

### Transfection of crRNA:tracrRNA duplex for arrayed CRISPR KO screening and generation of stable KO clones

All predesigned guidance RNAs (Edit-R synthetic crRNA), the non-targeting control#1 (Cat# U-007501-01-2), trans-activating CRISPR RNA (tracrRNA, Cat# U-002005-5000), as well as the DharmaFECT 1 transfection reagent (CAT# T-2001-04) were purchased from Horizon Discovery. crRNA and tracrRNA were diluted as a 10 μM stock solution in Tris buffer (pH 7.4). Co-transfection of the crRNA:tracrRNA duplex was performed as previously published (19, 31). IniDC_Cas9 cells were seeded in 6-well plates at 1x10^6^ / well in 2 mL DC medium without Dex/Dox. For each transfection, 25 nM crRNA and 25 nM tracrRNA were mixed in 100 μl RPMI1640 medium and incubated 5 min at room temperature; 10 μl of DharmaFECT 1 transfection reagent was mixed in 100 μl RPMI1640 medium and incubated 5 min at room temperature; then the two solutions were mixed and incubated for another 20 min before being added dropwise into iniDC_Cas9 cultures. Three days after transfection the cells were collected for FACS sorting to obtain clones carrying the KO of interest. For genetic screening purposes, the transfection reagent containing medium was replaced with fresh DC medium to let the cells recover overnight before the *in vitro* antigen cross-presentation assay.

### In vitro antigen cross-presentation assay

BMDCs or de-iniDCs and their derivates carrying specific gene knockouts, or de-iniDCs that were transiently transfected with crRNA:tracrRNA for screening, were collected by trypsinization and diluted to 5x10^5^ cells/mL with DC medium containing β-mercaptoethanol and GM-CSF. One hundred μL cell suspension/well was seeded in 96-well tissue culture U-bottom plates (equal to 5x10^4^ cells/well). The cells were treated or not with BCL2 family inhibitors as detailed in the figure legends. Then soluble OVA was added into the cell culture at a final concentration of 1 mg/mL and incubated for 4 h at 37°C and 5% CO_2_. The plates were then centrifuged at 500 g for 5 min, supernatant was removed and replaced with 200 μL/well of RMPI 1640 medium. This step was repeated once to wash out the remaining OVA. B3Z T cell hybridomas were collected by centrifugation of the suspension culture and diluted to 5x10^5^ cells/mL with DC medium. After removing the supernatant from DCs washed twice, 200 μL/well of B3Z suspension was added, and co-incubated with DCs for 18 h at 37°C before collecting the supernatant by spinning the plates at 500 g for 5 min and gently transferring 150 μL supernatant for the quantification of IL2 secretion by ELISA.

### Customized ELISA

ELISA for IL1β, IL2, and IL6 was performed as previously published (19). In brief, capture antibody was diluted in 1 x ELISA coating buffer (diluted with water from 5x ELISA coating buffer obtained from Biolegend) at 1/500, applied 100 μL/well in 96-well high-binding assay plates (Corning), and incubated overnight at 4°C; then the plates were washed 3 times with washing buffer (1x TBS with 0.1% Tween-20, 300 μL/well) and incubated with 150 μL/well blocking buffer (10% FBS + 1% BSA in PBS) for 1 h at room temperature to block unspecific binding sites. Then plates were loaded with samples or serially-diluted standards and incubated for 2 h at room temperature. Afterwards the supernatant was discarded and plates were washed 4 times with washing buffer, then 100 μL/well of biotinylated detection antibody (1/500 diluted in blocking buffer) was added and incubated at room temperature for 1 h. The supernatant was discarded and plates were washed 4 times with washing buffer, then 100 μL/well of HRP-Avidin (Cat# 405103 from Biolegend, 1/1000 diluted in blocking buffer) was added and incubated at room temperature for 30 min. In the end, plates were washed 5 times and 100 μL/well of 1-Step™ Ultra TMB-ELISA substrate solution (Cat# 34028 from Life Technologies) was added for colorization. When the top standard wells turned dark blue (generally within 10 min), 50 μL/well of 0.5 M H_2_SO_4_ was used to stop the reaction. The absorbance at 450 nm was immediately measured using a BMG FLUOstar plate reader. Exact concentration of the assayed cytokine was calculated by the standard curve and corresponding dilution factors of the sample.

### RNA-seq and analysis

Total RNA was extracted from cultured de-iniDCs (~5x10^7^ cells) with the RNeasy Plus Mini Kit (Qiagen) following the manufacturers’ instructions. RNA-Seq data analysis was performed by GenoSplice technology (www.genosplice.com, Paris, France). Analysis of sequencing data quality, reads repartition (e.g., for potential ribosomal contamination), inner distance size estimation, gene body coverage, strand-specificity of library were performed using FastQC v0.11.2, Picard-Tools v1.119, Samtools v1.0, and RSeQC v2.3.9. Reads were mapped using STAR v2.4.0f1 (55) on the mouse mm10 genome assembly and read count was performed using featureCount from SubRead v1.5.0. Gene expression was estimated as described previously (56) using Human FAST DB v2018_1 annotations. Only genes expressed in at least one of the two compared conditions were analyzed further. Genes were considered as expressed if their FPKM value was greater than FPKM of 97% of the intergenic regions (background). Analysis at the gene level was performed using DESeq2 (57) using experiment ID in the DESeq2 GLM model. Genes were considered differentially expressed for fold-changes ≥1.5 and p-values ≤0.05. Pathway analyses were performed using WebGestalt v0.4.4 (58) merging results from upregulated and downregulated genes only, as well as all regulated genes. Pathways and networks were considered significant with p-values ≤0.05.

### Mouse scRNA-seq Data Analysis

We utilized publicly available raw 10x single-cell transcriptomic data from Brown et al. 2019 (59), downloaded from the GEO repository (GSE137710). Using provided metadata, we selected annotated cells and reprocessed data using Seurat’s SCTransform function with default parameters. We then performed dimension reduction using RunPCA and RunUMAP with default parameters.

ssGSEA was performed using the GSVA R package with gene signatures from the scRNAseq dataset published by Brown et al. 2019 that were obtained through FindAllMarkers Seurat’s function. For broader myeloid cell types the SiglecH DC cluster was considered as pDC and cDC2 Tbet^+^, cDC2 Tbet^-^, cDC2 mixed, cDC1, CCR7hi DC clusters were gathered as cDC mega cluster. For refined DC cell types, cDC2 Tbet^+^, cDC2 Tbet^-^, cDC2 mixed clusters were merged as cDC2.

We deconvoluted our bulk RNA-seq with the DC Mouse scRNA-seq dataset using BayesPrism (60) following the standard pipeline to exclude outliers and align based on protein coding genes. Plots were generated in R using the ggplot package.

### Immunoblotting

The entire immunoblotting procedure was performed according to the standard protocol of the NuPAGE^®^ electrophoresis system (Invitrogen) and all reagents were purchased from Life Technologies if not otherwise specified. Protein extracts were obtained by lysing cells in RIPA buffer containing a protease inhibitor cocktail, 1 x SDS Loading Buffer, and 1x Sampling Reducing Buffer. Then proteins were separated on 4–12% NuPAGE® Bis-Tris gels in the NuPAGE® MES SDS Running Buffer and electro-transferred to 0.45 μm polyvinylidene fluoride (PVDF) membranes (Bio-Rad, Hercules, CA, USA) in 1 x Tris-Glycine (TG) buffer. Membranes were incubated for 1 h in 5% BSA dissolved in TBST (Tris-buffered saline containing 0.05% Tween 20) to block unspecific binding sites, followed by incubation with the primary antibody overnight at 4°C. Membranes were then washed 5 times with TBST and incubated with horseradish peroxidase (HRP)-conjugated secondary antibodies (Southern Biotechnologies, Birmingham, AL, USA), for 2 h at room temperature. After 5 times washing with TBST, blots were subjected to chemiluminescence-based detection using the Amersham ECL Prime kit, on a ImageQuant LAS 4000 imager (GE Healthcare, Piscataway, NJ, USA). Quantitation of chemiluminescence signals of bands of interest was performed with the integrated software ImageQuant TL.

### Immunostaining and fluorescence microscopy

Wild-type or *Bcl2*^-/-^ de-iniDCs were seeded in Poly-D-Lysine-treated 96-well Assay Plate (Corning® BioCoat®) and let adapt overnight. Upon treatment with venetoclax for 24h cells were stained with the MitoTracker™ Orange CMTMRos dye (1/4,000 diluted in serum-free RMPI1640 medium) for 30 mins, and then washed once with PBS before fixing with 10% paraformaldehyde containing 5 μg/mL Hoechst 33342 for 20 mins at room temperature. Cells were then washed twice with PBS, permeabilized with 0.1% Triton X-100/PBS for 10 mins, and blocked with 1% BSA/PBS for 1 hour before incubated with a mouse-anti-double strain DNA antibody (dsDNA, 1/200 diluted in 1% BSA/PBS) at 4-degree overnight. Then cells were washed twice with PBS and incubated with AlexaFluor® 488 anti-mouse secondary antibody (diluted 1/250 in 1% BSA/PBS) for 45 min at room temperature. Cells were then washed once with PBS and the plate were sealed with adhesive aluminum for image acquisitions using an ImageXpress Micro C automated confocal microscope (Molecular Devices, Sunnyvale, California, USA) equipped with a 20× PlanApo objective (Nikon, Tokyo, Japan). A built-in Custom Module Editor from MetaXpress Software was used for image analysis. Cytoplasmic and nuclear regions of interest (ROI) were segmented and the mitochondrial region was defined by MitoTracker™ Orange staining. Fluorescence intensities, dots count, and dots area were measured within these defined regions. Cell-based measurements were transformed into well-based data with the Microsoft Excel Pivot table, which was then graphically depicted and statistically evaluated using the GraphPad Prism software. A minimum of 4 view fields/well was acquired and analyzed.

### Apoptosis assay

Cell death was assessed by means of the FITC-Annexin V/DAPI staining protocol. Cells were treated in 6-well plates as detailed in the figure legends, collected by trypsinization and washed in PBS before the cell pellet was resuspended in 50 μL Annexin V binding buffer containing FITC-conjugated Annexin V (both from Biolegend). Samples were then incubated in the dark for 15 min before addition of 400 μL staining buffer supplemented with 1 μg/mL DAPI. Acquisitions were performed on a BD Fortessa cytofluorometer, and data were analyzed and statistically evaluated using FlowJo.

### Quantitative RT-PCR

RNA extraction from bone marrow cells and spleenocytes was performed with the GeneJET RNA Purification Kit (Life Technologies). following the manufacturers’ instructions. Reverse transcription from mRNA to cDNA was performed with the Maxima First Strand cDNA Synthesis Kit (Life Technologies), using approximately 2.5 μg total RNA as template. Real-time PCR reaction was performed on a StepOnePlus Real-Time PCR System (Applied Biosystems, Foster City, CA, USA) using the Power SYBR™ Green PCR Master Mix and corresponding settings. Gene-specific primers were designed by using the NCBI Primer-BLAST online application (https://www.ncbi.nlm.nih.gov/tools/primer-blast/) and synthesized by Eurofins Genomics. Primer sequences are listed in Table S4. qRT-PCR data was analyzed using the 2-^ΔΔCt^ method to obtain the fold change in gene expression that were normalized to expression levels of the housekeeping gene *Gapdh*.

### Animals and cancer models

All mice with the TC1 orthotopic lung cancer model and MCA205 orthotopic fibrosarcoma model were maintained at the Gustave Roussy Campus Cancer in a specific pathogen free (SPF), environmental controlled animal facility with 12 h light/dark cycles, receiving food and water *ad libitum*. All animal experiments were performed in compliance with the EU Directive 63/2010 and dedicated ethic protocols (Projects 2020_036 and 2021_010) that was approved by the ethical committee of the Gustave Roussy Campus Cancer, CEEA IRCIV/IGR no. 26, registered at the French Ministry of Research). Female wild-type C57BL/6 mice (6~8 eight weeks old) and female athymic nude (*nu/nu*) mice were obtained from ENVIGO France (Gannat, France). The *Batf3-KO* mice were maintained in the animal facility of University Hospital Erlangen. Bone marrow from WT or *Batf3-KO* mice were flushed from femur and tibia, dissociated into single cells and cryopreserved at -80°C. For bone marrow transplantation, the cells were thawed in a 37°C water bath, washed with worm PBS containing 5% FBS, and resuspended in cold PBS. Five million bone marrow cells were engrafted into lethally irradiated (10 Gy) congenic recipient mice, which were maintained and monitored for two months before being used for tumor establishment. Orthotopic fibrosarcoma and NSCLC models were established as previously published (61). For the fibrosarcoma model, 5x10^5^ wild type MCA205 cells were *s.c*. inoculated into the right flank of mice, which were randomly assigned into treatment groups (n = 6–8 animals per group). When tumors became palpable (surface, calculated as longest dimension × perpendicular dimension × π/4, around 20–25 mm^2^), mice received the treatments described below. Tumor surface was then regularly monitored and animals bearing neoplastic lesions that exceeded 250 mm^2^ were euthanized. For the TC1 NSCLC model, wild type TC1 Luc cells (5x10^5^ in 100 μL PBS) were intravenously injected to mice. Tumor incidence and development were monitored by *in vivo* photonic imaging of tumor cell luciferase activity. When tumor incidence in the lung was detected at an exposure time of 4 min (6~7 days after cell injection), mice were randomized for treatment as described below. To perform bioluminescence imaging, mice were injected *i.p.* with 3 mg beetle luciferin potassium salt dissolved in DPBS (Promega, Madison, WI, USA), After 8 min (at peak bioluminescence signal) mice were anesthetized with vaporized isoflurane and photons were acquired on an IVIS Lumina III imaging system (Caliper Life Sciences Inc., Hopkinton, MA, USA). *In vivo* imaging was conducted every 4–5 days with an exposure time starting with 4 min gradually decreased to 1 min when photon saturation occurred. Tumor bearing mice showing photon saturation at 1 min of exposure at small binning settings were euthanized.

Mice for the spontaneous breast cancer model were housed in the SPF animal facility of Weill Cornell Medical College, and all experimentation was aligned with the Guidelines for the Care and Use of Laboratory Animals guidelines and approved by the Institutional Animal Care and Use Committee (IACUC) (no. 2023-0014). 6–9-week-old female C57BL/6J mice (Taconic Bioscience) were subcutaneously implanted with 50 mg slow-release (90 d) medroxyprogesterone acetate (MPA, M) pellets (Innovative Research of America) followed by oral gavage with 1 mg 7,12-dimethylbenz[a]anthracene (DMBA, D) in 200 μL corn oil once a week for 7 weeks after pellet implantation (62). Mice were then routinely assessed for the development of M/D-driven malignant lesions along the mammary lines, until reaching a surface area of 12–25 mm^2^ (d0). Mice bearing M/D-driven mammary tumors were randomly allocated to treatments included (1) vehicle control: 100 μL PEG delivered oral gavage daily on d0 until the end of the experiment; (2) venetoclax: 100 mg/Kg delivered oral gavage in 100 μL vechicle on d0 until the end of the experiment; (3) focal RT: three fractions of 10 Gy each (total dose: 30 Gy, dose rate: 271 cGy/min) delivered to the primary tumor on d0, d1 and d2; (4) focal RT followed by venetoclax; (5) focal RT followed by venetoclax and anti-IFNAR-1 antibody (Clone MAR1-5A3, from BioXcell) delivered *i.p.* at 20 mg/Kg on d-1 and then weekly until the end of the experiment. Mice were routinely assessed for the emergence of toxicity (troublesome breathing, weight loss, anorexia, hunched posture) and tumor growth. Mice bearing M/D-driven tumors were euthanized when tumor burden area reached 180–200 mm^2^.

### Adoptive DC transfers and detection in vivo

IniDC_Cas9 cells or derivates carrying gene-KO were cultured in medium without Dex/Dox to be differentiated into de-iniDCs as described before. Three days after withdrawing Dex/Dox, the lysates prepared from equal numbers of MCA205 or TC1-Luc cells (by freezing–thawing process in liquid nitrogen and a water bath, followed by sonication to ensure complete disruption of cells) was added to the cell culture and incubated for 2 h for tumor antigen exposure. In some cases, the 3-day differentiated de-iniDC_Cas9 cells were pretreated with navitoclax or venetoclax at 5 μM for 4 h before being exposed to cancer cell lysates. Then the antigen-loaded de-iniDC_Cas9 cells were collected by trypsinization or by scrapping the cell culture layer with a plastic cell lifter. Cells were washed twice with cold PBS and passed through a 70 μm strainer to remove clogs. The single cell suspension was diluted in cold PBS for *intertumoral* injection (1x10^6^ cells/mouse) or *i.v.* injection (2x10^6^ cells/mouse). Where indicated, the cells were incubated with an IFNAR-blocking monoclonal antibody at 10 μg per 1x10^6^ DCs, or with an equal quantity of isotype control antibody for 30 mins before *i.v.* injection. To monitor the migration of de-iniDCs, cells were stained with the long-lasting red fluorescent dye PKH26 following the manufacture’s protocol. After injection, the lung and lung cancer-draining mediastinal lymph nodes were excised at different time points and digested to single cell suspensions for multiplex immunofluorescence staining and flow cytometric analysis, which allowed for subgating of DC markers and PKH26 red fluorescence. Alternatively, after i.v. injection of de-iniDCs the complete lung was excised together with the trachea, flushed with cold PBS, and then quickly infused with 4% PFA (diluted with PBS) via the trachea, which were then closed with surgical suture and immersed into a large volume of 4% PFA. Fixed lungs were embedded in paraffin and subjected to tissue sectioning and immunohistochemistry.

### Chemical and antibody treatment in vivo

Solvent (Sol) for chemicals is formulated as 10% Tween-80, 10% PEG400, and 4% DMSO in physiological saline. Navitoclax and venetoclax were administrated *i.p.* at a dose of 50 mg/Kg, following the schedule specified in the figures and corresponding legends. In case of combination with checkpoint blockade, mice received *i.p.* injection of either 200 μg anti-PD-1 antibody, 100 μg anti-CTLA-4 antibody, or 200 μg isotype antibody, at 8, 12 and 16 days after the first chemical treatments. For T cell depletion, mice received *i.p.* injections of 100 μg anti-CD8 plus 100 μg anti-CD4 antibody, or 200 μg of isotype antibody, one day before and the same day of pharmacological treatment or cell transfer, which were continued at a frequency of once a week for two weeks. For CD11b neutralization, mice received 100 μg anti-CD11b or equal amounts of isotype control antibody as scheduled for anti-CD4/CD8, but were treated every other day for the following 2 weeks. In some cases, mice were treated *i.v.* with 5 mg cytochrome C/mouse in PBS or PBS alone following the same schedule as anti-CD11b.

### Tissue dissociation and flow cytometry staining

Orthotopic MCA205 fibrosarcoma or TC1 NSCLC cancers were established and tumor bearing mice were treated as described above. At day 3 or day 7 after treatment, blood was collected from tumor bearing mice via cardiac puncture (under anesthesia with vaporized isoflurane) into 2 mL centrifuge tubes containing EDTA-K. Then mice were euthanized for excising tumors and immune organs. The samples were collected in cold RPMI-1640 medium and kept on ice until dissociation. Blood was directly subject to erythrocyte elimination by using 1x red blood cell lysing buffer (Biolegend); spleen and lymph nodes were squeezed through 70 μm strainers (Corning) with the rubber tip of 1 mL syringe to generate single cell solutions; tumor-bearing lungs and excised *s.c*. tumors were digested in an enzymic buffer containing 1mg/mL collagenase type IV (Life technologies) and DNase I (Sigma). The dissociated bulk cell suspension was resuspended in RPMI-1640, passed through 70 μm cell strainers and washed twice with cold PBS. Cells from spleen and lung were further treated with 1x red blood cell lysis buffer to remove erythrocytes. Prior to surface staining of fluorescent antibodies, samples were incubated with LIVE/DEAD^®^ Yellow Fixable dye to label damaged/dead cells, and incubated with antibodies against CD16/CD32 to block Fc receptors. For multiplex staining, cells were incubated with a panel of fluorescence-conjugated antibodies for 30 min surface staining in the dark. In the case of Foxp3 staining, the surface-labeled cells were permeabilized and fixed using a Foxp3/Transcription Factor Staining Buffer kit (Life Technologies), and stained with the FOXP3 FITC antibody for another 30 min. Otherwise, surface-labeled cells were directly fixed with 4% PFA (Sigma). After 2 times wash, the cells were kept at 4°C until flow cytometric analysis. For the intracellular staining of IL12 p40, the dissociated cells were incubated with Brefeldin A (5 μg/mL diluted in RPMI medium) for 2 hours before surface staining. The surface-labeled cells were permeabilized and fixed using a Foxp3/Transcription Factor Staining Buffer kit, and stained with the IL12_APC antibody for another 30 min. Data were acquired on a BD LSRFortessa flow cytometer (BD Biosciences) and analyzed using the FlowJo software. Detailed gating strategies for the flow cytometric analysis are provided in corresponding Supplementary Figures.

### Clinical Specimens and Human PBMCs

Peripheral blood samples were collected from patients with AML, before and after treatment with venetoclax in combination with azacytidine, at the Gustave Roussy Cancer Institute. Written informed consent authorizing the blood samples to be used for research purpose was obtained from all patients, and the study was approved according to the guidelines of the protocol alpha PPP ID RCB: 2020 A03290-39 by the review board of Gustave Roussy Cancer Institute. Peripheral blood was also collected from healthy donors aged 25-35. Peripheral blood mononuclear cells (PBMCs) were isolated by Ficoll-Paque PLUS™ (GE Healthcare) density gradient centrifugation following the manufacturer’s protocol. PBMCs from AML patients were cryopreserved at -80°C until staining. PBMCs from healthy donors were treated *in vitro* with venetoclax, azacytidine alone or their combination (both at a concentration of 5 μM) for 18 h, and then cryopreserved as well before being stained together with the patients’ PBMCs.

For multiplex staining, frozen PBMCs were rapidly thawed in a 37°Cwater bath, pooled in 20 ml RPMI 1640 medium, and centrifuged at 500 g for 5 min at 4°C. After discarding supernatant, cells were resuspended in 1 mL cold PBS and transferred to 5 mL FACS tubes for additional steps. After centrifugation and removal of supernatant, the cell pellets were resuspended with 500 μL of cold viadye red solution (1/500 diluted in PBS) and incubated at 4°C for 15 min. Then 10 μL of FBS was added to each sample (equals to 2% FBS) and the cells were incubated for another 15 min. During this period, the antibody cocktail was prepared in two staining batches, both in the Brilliant Stain buffer (BD Bioscience) containing 2% FBS and 2 mM EDTA. Batch #1 contains CCR2_BUV563, CD169_BUV661, SLAN_VioBlue, CD25_BV605, CX3CR1_BV711, PD-1_BV750, XCR1_FITC, CD206_PP/eFluor710, CCR7_BB755, CD80_PE, PD-L1_PE/Cy5, CD40_PE/Cy7, CD178_APC, CADM1, CD5_APC/R700, CD88_APC/Fire750, CD8_APC/Fire810. Batch #2 contains AlexaFluor647 goat-anti-chicken 2^nd^ antibody (for CADM1), HLA-DQ_BUV395, CD38_BUV615, CD11c_BUV805, CD141_BV421, CD1c_SB436, CD83_BV510, CD3/16/19/20_BV650, CD86_BV786, CD14_SparkB550, CD45_PercP, CD4_Cfl.YG584, CD123_PE/Daz594, CD69_PE/Cy5.5, HLA-DR_PE/Fire810, and CD45RA_Spark NIR685. Following Viadye red staining, 2 mL of FACS buffer was added into each tube and cells were spun down. The cells were then resuspended in the batch #1 antibody cocktail (100 μL/sample) and incubated at 37°C for 30 min. Then 2 mL FACS buffer was added and the supernatant was removed after centrifugation. Pellets were resuspended in the batch #2 antibody cocktail and incubated for 30 min at 4°C. After the 2^nd^ staining, the cells were spun down and washed twice with 2 mL FACS buffer. The stained cells were finally resuspended in 500 μL FACS buffer and acquired on a Cytek Aurora flow cytometer with compensations. Data analysis were performed with the FlowJo software.

### Statistical analysis

Statistical significance was calculated using the Graphpad Prism software (Version 9.0.2), by means of one-way or two-way ANOVA test (with FDR or Dunnett’s multiple comparisons test), unpaired or paired Student t test, or Fisher’s exact test, as detailed in the corresponding figure legends. *TumGrowth* was used to analyze *in vivo* data (63): linear or log-transformed mixed-effects models for longitudinal comparison of tumor growth curves by type II ANOVA; cross-sectional analysis with Likelihood ratio test for comparing endpoint tumor size distribution; and Cox proportional hazards regression or logrank test for comparing survival curves. *TumGrowth* is free available at Github/Kroemerlab. P values of 0.05 or less were considered to denote significance and were properly annotated in the figures.

## Supplementary Material

Figure S1

Figure S2

Figure S3

Figure S4

Figure S5

Figure S6

Figure S7

Figure S8

Figure S9

Figure S10

Supplementary table legends

Table S1

Table S2

Table S3

Table S4

## Data Availability

CRISPR screen data generated is provided in Table S1. Results from arrayed KO screens are provided in Table S2. The bulk RNA-seq data has been deposited in the Gene Expression Omnibus (GEO) database under accession numbers GSE218062. All other raw data generated or analyzed during this study are available upon request from the corresponding authors. No code or programs have been generated in this study.

## Significance statement

BCL2 inhibition improves the capacity of DCs to stimulate anticancer immunity and restrain cancer growth in an immunocompetent context but not in mice lacking cDC1 or mature T cells. This study indicates that BCL2 blockade can be employed to sensitize solid cancers to PD-1/PD-L1-targeting immunotherapy.
