# Supplementary material for: BCL2 inhibition reveals a dendritic cell-specific immune checkpoint that controls tumor immunosurveillance
Source: Cancer Discov. Author manuscript; Available in PMC 2023 Nov 1. (PMC7615270; doi:10.1158/2159-8290.CD-22-1338)
Supplement: Figure S1 [file EMS187151-supplement-Figure_S1.pdf]

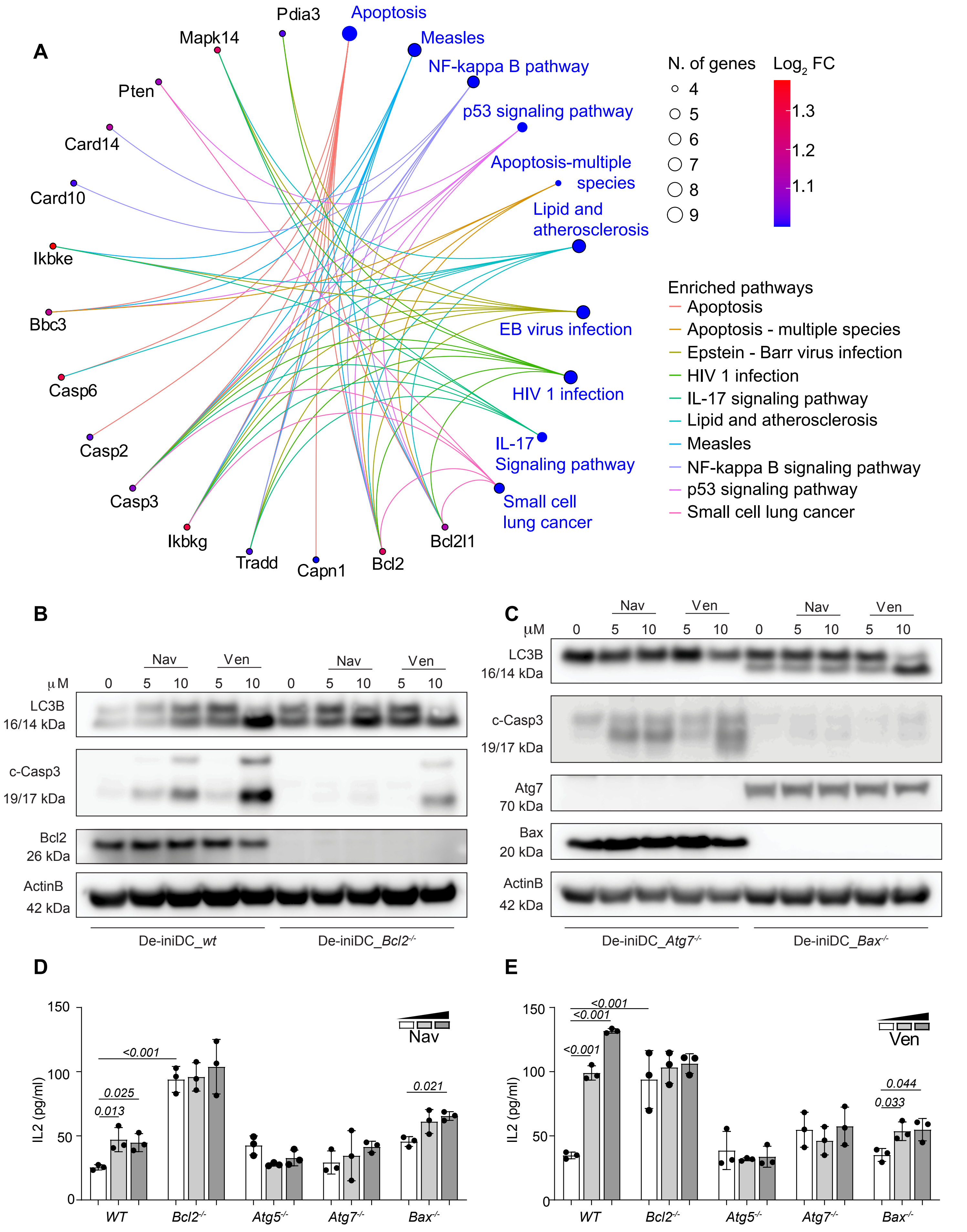
A
Pdia3
Mapk14
Pten
Card14
Card10
Ikbke
Bbc3
Casp6
Casp2
Casp3
Ikbkg
Tradd
Capn1
Bcl2
Bcl2l1
Apoptosis
Measles
NF-kappa B pathway
p53 signaling pathway
Apoptosis-multiple species
Lipid and atherosclerosis
EB virus infection
HIV 1 infection
IL-17 Signaling pathway
Small cell lung cancer
N. of genes
4
5
6
7
8
9
Log2 FC
1.3
1.2
1.1
Enriched pathways
Apoptosis
Apoptosis - multiple species
Epstein - Barr virus infection
HIV 1 infection
IL-17 signaling pathway
Lipid and atherosclerosis
Measles
NF-kappa B signaling pathway
p53 signaling pathway
Small cell lung cancer
B
Nav
Ven
Nav
Ven
μM 0 5 10 5 10 0 5 10 5 10
LC3B 16/14 kDa
c-Casp3 19/17 kDa
Bcl2 26 kDa
ActinB 42 kDa
De-iniDC_wt
De-iniDC_Bcl2-/-
C
Nav
Ven
Nav
Ven
μM 0 5 10 5 10 0 5 10 5 10
LC3B 16/14 kDa
c-Casp3 19/17 kDa
Atg7 70 kDa
Bax 20 kDa
ActinB 42 kDa
De-iniDC_Atg7-/-
De-iniDC_Bax-/-
D
IL2 (pg/ml)
150
100
50
0
Nav
<0.001
0.025
0.013
0.021
WT
Bcl2-/-
Atg5-/-
Atg7-/-
Bax-/-
E
IL2 (pg/ml)
150
100
50
0
Ven
<0.001
<0.001
<0.001
0.044
0.033
WT
Bcl2-/-
Atg5-/-
Atg7-/-
Bax-/-

Figure S1

Supplementary Figure S1. Pathways and mechanistic exploration of enhanced antigen cross-presentation by BCL2 inhibitors. (A) KEGG mapping with the top candidates from the arrayed screening. The cnetplot depicts the connections of the top 15 genes and enriched KEGG pathways (top 10 according to enrichment factor). (**B, C**) De-iniDC_Cas9 stably knocked out for *Bcl2, Atg5, Atg7, or Bax* were treated with navitoclax (Nav) or venetoclax (Ven) at 0 (DMSO), 10, or 20 μM, for 10 h to measure the induction of autophagy and apoptosis by immunoblot, or overnight with lower concentrations (5, 10 μM) for the evaluation of antigen cross-presentation as indicated by the secretion of IL2 (**D, E**), and displayed as bar chart (mean ± SD, n = 3). Statistical significance was calculated by means of the Student *t* test with Bonferroni correction. P-values are labelled in the figure to indicate significance of difference.
