# Supplementary material for: BCL2 inhibition reveals a dendritic cell-specific immune checkpoint that controls tumor immunosurveillance
Source: Cancer Discov. Author manuscript; Available in PMC 2023 Nov 1. (PMC7615270; doi:10.1158/2159-8290.CD-22-1338)
Supplement: Figure S2 [file EMS187151-supplement-Figure_S2.pdf]

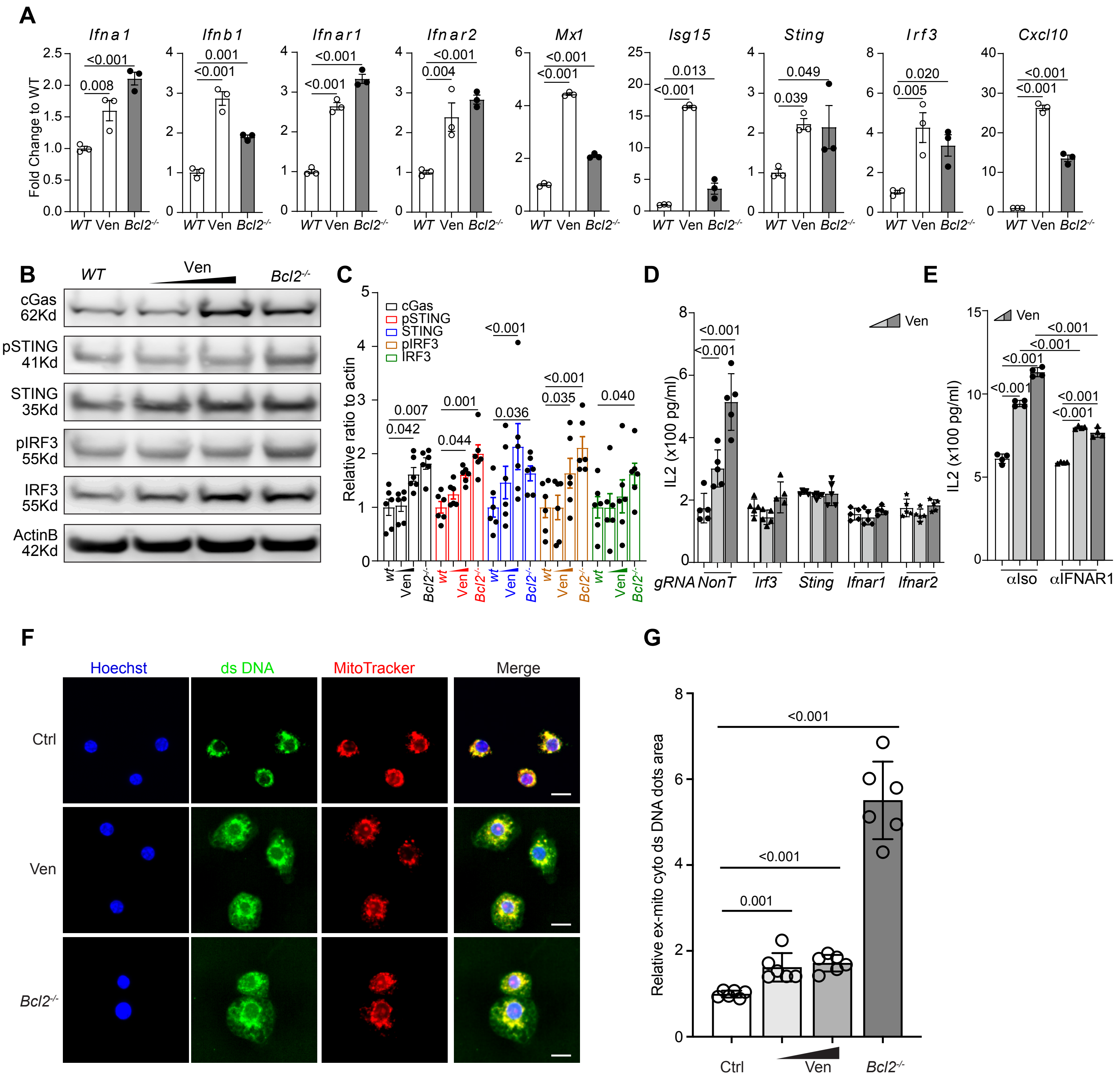

A
Ifna1
Ifnb1
Ifnar1
Ifnar2
Mx1
Isg15
Sting
Irf3
Cxcl10
Fold Change to WT
WT Ven Bcl2-/-
B
WT
Ven
Bcl2-/-
cGas 62Kd
pSTING 41Kd
STING 35Kd
pIRF3 55Kd
IRF3 55Kd
ActinB 42Kd
C
cGas
pSTING
STING
pIRF3
IRF3
Relative ratio to actin
D
IL2 (x100 pg/ml)
gRNA NonT Irf3 Sting Ifnar1 Ifnar2
E
IL2 (x100 pg/ml)
αIso αIFNAR1
F
Hoechst
ds DNA
MitoTracker
Merge
Ctrl
Ven
Bcl2-/-
G
Relative ex-mito cyto ds DNA dots area
Ctrl
Ven
Bcl2-/-

Figure S2

Supplementary Figure S2. Chemical inhibition or genetical invalidation of Bcl2 activates type I interferon signaling. (**A-C**) Wild type (WT) de-iniDC were left untreated or treated with venetoclax (Ven, 5 or 10 µM) for 48 hours and subjected to total RNA extraction for quantitative PCR (**A**) or protein extraction for western blots (**B**). RNA and protein extracted from *Bcl2*$^{-/-}$ de-iniDCs were analyzed together. Signals emitted by protein band(s) of interest were quantified and normalized to beta-actin (ActinB) to generate a scattered dots plot (**C**, mean ± SD, n = 6 replicates). (**D**) IniDC_Cas9 cells were transfected with gRNAs targeting *Irf3, Sting, Ifnar1, Ifnar2,* or non-targeting control gRNA (NonT) to establish transient knockouts and simultaneously differentiate the precursors into de-iniDC_Cas9, which were then treated with Ven overnight. (**E**) Alternatively, de-iniDCs were pretreated with an IFNAR1-blocking antibody (αIFNAR) or isotype control antibody (αIso) before adding Ven into the system. The treated cells were subjected to *in vitro* antigen cross-presentation assays. IL-2 production was quantified by ELISA and reported as scattered dots plot (mean ± SD, n = 5 or 4 replicates). (**F,G**) WT de-iniDCs, were left untreated or treated with Ven for 24h, and *Bcl2*$^{-/-}$ de-iniDCs were subjected to immunofluorescence staining of double stranded DNA (dsDNA, green dots). MitoTracker orange (red) and Hoechst 33342 (blue) were used to mark mitochondria and nuclei, respectively. Representative images of different treatment conditions are displayed in **F**, scale bars equal 10 µm. The areas of dsDNA dots in the extra-mitochondrial cytoplasm (Ex-mito cyto) were quantified and normalized to the untreated WT condition (mean ± SD, n = 6) in **G.** Statistical significance was calculated by means of one-way ANOVA with Tukey's multiple comparisons test.
