# Supplementary material for: BCL2 inhibition reveals a dendritic cell-specific immune checkpoint that controls tumor immunosurveillance
Source: Cancer Discov. Author manuscript; Available in PMC 2023 Nov 1. (PMC7615270; doi:10.1158/2159-8290.CD-22-1338)
Supplement: Figure S3 [file EMS187151-supplement-Figure_S3.pdf]

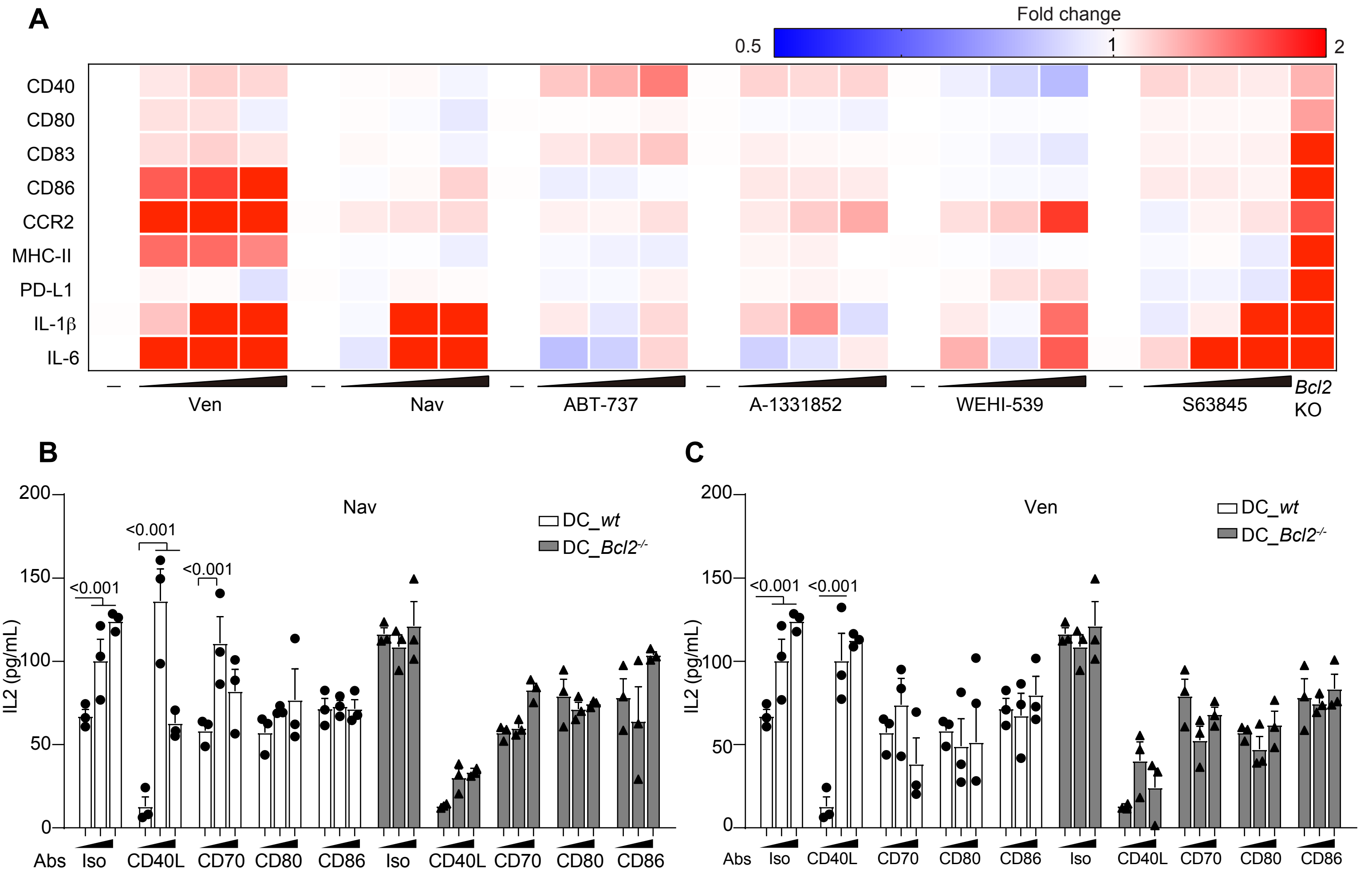
A
Fold change
0.5
1
2
CD40
CD80
CD83
CD86
CCR2
MHC-II
PD-L1
IL-1β
IL-6
Ven
Nav
ABT-737
A-1331852
WEHI-539
S63845
Bcl2 KO
B
Nav
DC_wt
DC_Bcl2-/-
IL2 (pg/mL)
<0.001
Abs
Iso
CD40L
CD70
CD80
CD86
C
Ven

Figure S3

Supplementary Figure S3. Chemical inhibition or genetical invalidation of Bcl2 enhances the activation and maturation of de-iniDCs. (**A**) WT de-iniDCs were treated with different Bcl2 family inhibitors overnight for flow cytometric analysis of DC activation and maturation markers, or for ELISA quantification of IL1β and IL6 in the cell culture supernatant. The mean fluorescence intensity (MFI) of surface markers and concentrations of cytokines were normalized to DMSO controls (-) and hierarchically clustered in a heatmap (mean, n = 3 or 4). (**B,C**) WT or *Bcl2*$^{-/-}$ de-iniDCs were pretreated with the indicated monoclonal blocking antibodies (Abs), or equivalent isotype control antibody, for 4 h before addition of Nav (**B**) or Ven (**C**) at 5 or 10 μM overnight for the evaluation of antigen cross-presentation. Secretion of IL2 was quantified and displayed as scattered dots blots (mean ± SD, n = 3). Statistical significance comparing with DMSO-treatment was calculated by means of one-way ANOVA with Turkey multiple comparisons test. P-values are labelled in the figure to indicate significance of difference.
