# Supplementary material for: BCL2 inhibition reveals a dendritic cell-specific immune checkpoint that controls tumor immunosurveillance
Source: Cancer Discov. Author manuscript; Available in PMC 2023 Nov 1. (PMC7615270; doi:10.1158/2159-8290.CD-22-1338)
Supplement: Figure S4 [file EMS187151-supplement-Figure_S4.pdf]

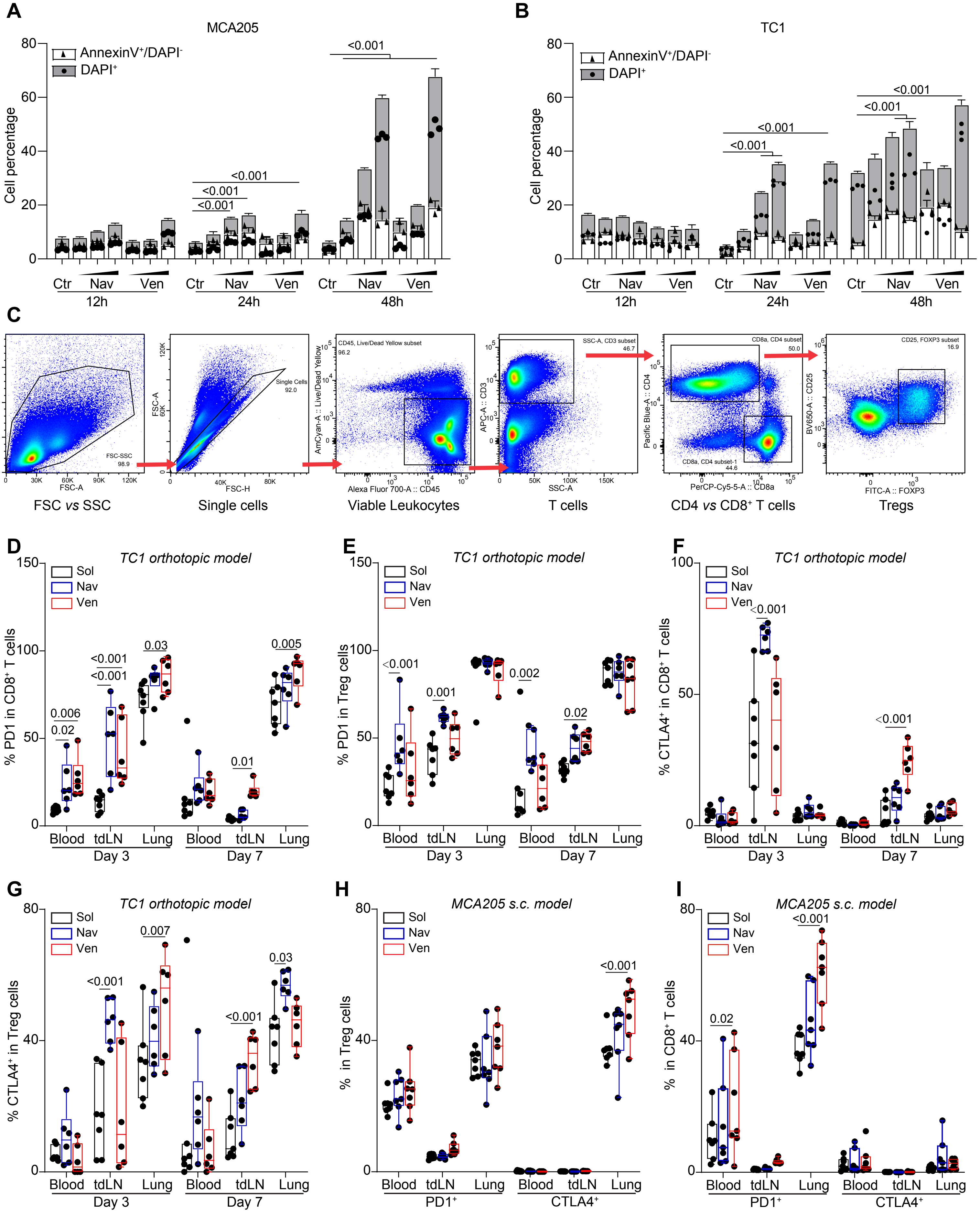
A
MCA205
AnnexinV+/DAPI-
DAPI+
Cell percentage
<0.001
Ctr
Nav
Ven
12h
24h
48h
B
TC1
C
FSC vs SSC
Single cells
Viable Leukocytes
T cells
CD4 vs CD8+ T cells
Tregs
D
TC1 orthotopic model
Sol
Nav
Ven
% PD1 in CD8+ T cells
Blood
tdLN
Lung
Day 3
Day 7
E
% PD1 in Treg cells
F
% CTLA4+ in CD8+ T cells
G
% CTLA4+ in Treg cells
H
MCA205 s.c. model
% in Treg cells
PD1+
CTLA4+
I
% in CD8+ T cells

Figure S4

Supplementary Figure S4. Impact of Bcl2 inhibitors on cancer cells and on the exhaustion of T cells. (A-B) Murine fibrosarcoma MCA205 and non-small cell lung cancer TC-1 cells were treated with increasing concentrations (5, 10, 20 μM) of navitoclax (Nav) or venetoclax (Ven) for the indicated time to evaluate apoptosis induction by means of annexin V-DAPI staining. Percentage of annexin V^{+}/$DAPI^{-}$ (represents apoptotic cells) and $DAPI^{+}$ (represents late apoptotic and necrotic cells) populations are depicted as stacked bar charts (mean ± SD, n=3). Statistical significance of non-healthy cells ($AnnexinV^{+}$ & $DAPI^{+}$ populations) as compared with DMSO controls was calculated by means of two-way ANOVA with Dunnett's multiple comparisons test. P-values are labelled in the figure to indicate statistical significance. (**C-I**) Orthotopic TC1 lung cancer- or MCA205 fibrosarcoma-bearing mice received two intraperitoneal (*i.p.*) injections of solvent (Sol), Nav, or Ven at day 0 (when bioluminescence detectable lung cancers or palpable MCA205 tumors appeared) and day 2. The indicated organs and blood were harvested at day 3 or Day 7 and dissociated into single cell suspensions for multiplex immunostaining and flow cytometric analysis of T cells. Gating strategies for the identification of T cell subsets is shown in (**C**). The percentage of PD-1^{+} or CTLA-4^{+} populations within different T cell subtypes is calculated with FlowJo and depicted as dot plots (n=6~7 animas/group) (**D-I**). Statistical significance was calculated using two-way ANOVA with Fisher's LSD test, as compared to Sol. P-values are labelled in the figure to indicate statistical significance.
