# Supplementary material for: BCL2 inhibition reveals a dendritic cell-specific immune checkpoint that controls tumor immunosurveillance
Source: Cancer Discov. Author manuscript; Available in PMC 2023 Nov 1. (PMC7615270; doi:10.1158/2159-8290.CD-22-1338)
Supplement: Figure S5 [file EMS187151-supplement-Figure_S5.pdf]

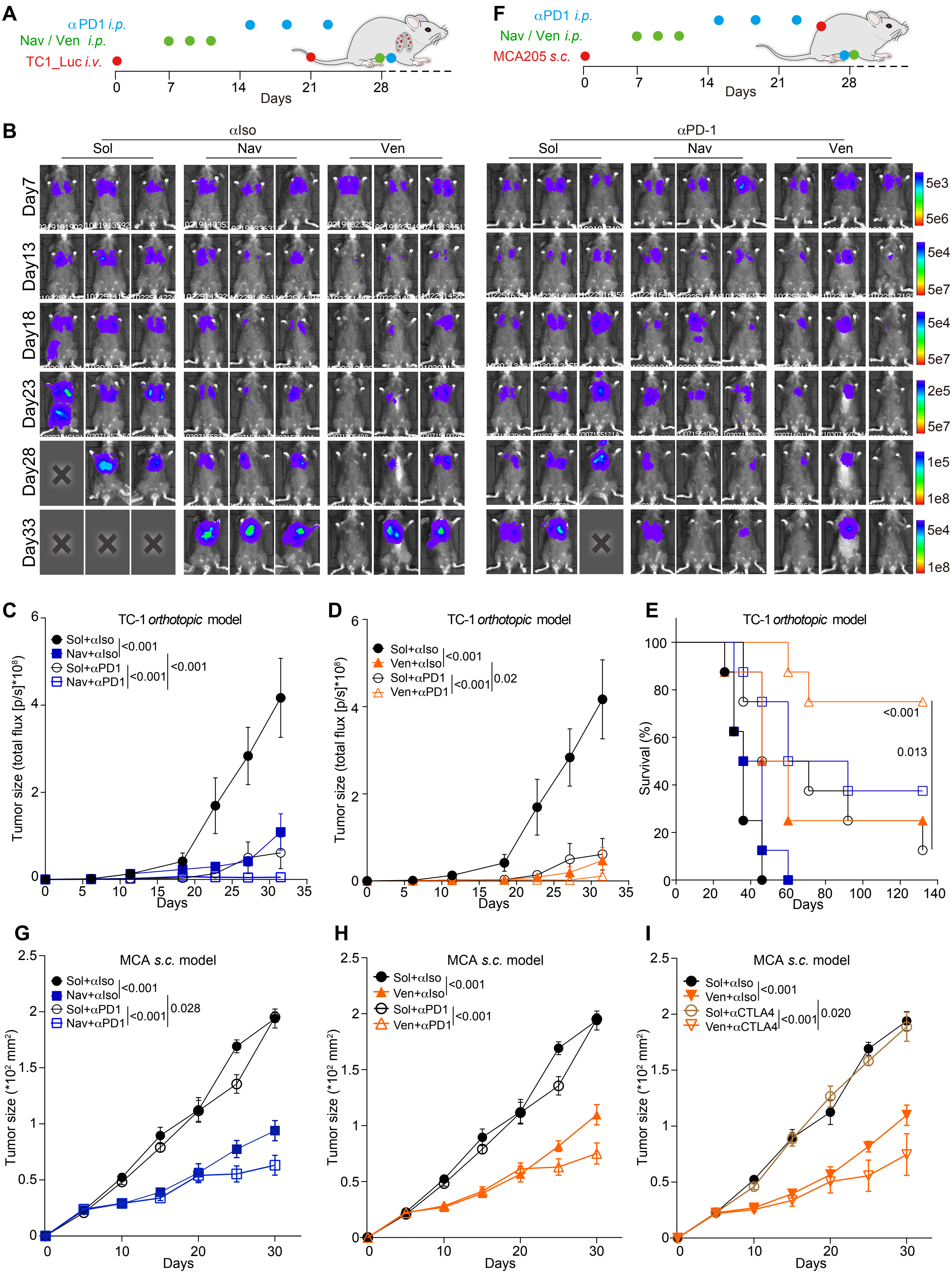

A
αPD1 i.p.
Nav / Ven i.p.
TC1_Luc i.v.
Days
F
αPD1 i.p.
Nav / Ven i.p.
MCA205 s.c.
Days
B
αIso
αPD-1
Sol
Nav
Ven
Day7
Day13
Day18
Day23
Day28
Day33
C
TC-1 orthotopic model
Sol+αIso
Nav+αIso
Sol+αPD1
Nav+αPD1
<0.001
Tumor size (total flux [p/s]*10^8)
Days
D
TC-1 orthotopic model
Sol+αIso
Ven+αIso
Sol+αPD1
Ven+αPD1
<0.001
0.02
E
TC-1 orthotopic model
Survival (%)
<0.001
0.013
G
MCA s.c. model
Sol+αIso
Nav+αIso
Sol+αPD1
Nav+αPD1
<0.001
0.028
Tumor size (*10^2 mm^2)
H
MCA s.c. model
Sol+αIso
Ven+αIso
Sol+αPD1
Ven+αPD1
<0.001
I
MCA s.c. model
Sol+αIso
Ven+αIso
Sol+αCTLA4
Ven+αCTLA4
<0.001
0.020


**Figure S5**

**Supplementary Figure S5. Systemic treatment of Bcl2 inhibitors sensitizes NSCLC tumors and fibrosarcoma to checkpoint blockades.** (**A**) TC1 lung cancer -bearing mice received three *intraperitoneal* (*i.p.*) injections of solvent (Sol), navitoclax (Nav), or venetoclax (Ven) at day 7 (when bioluminescence detectable lung cancers appear), day 9, and day 11. Subsequent treatments with blocking antibody to PD-1 (αPD-1), CTLA-4 (αCTLA-4), or corresponding isotype control antibody (αIso) were applied. (**B-E**) The orthotopic lung cancers were monitored by bioluminescence signals (**B**) which were quantified as total flux of photons. Tumor sizes development over time is depicted as mean ± SEM (n = minimum of 7 animals/group) (**C, D**). Overall survival was recorded and depicted as Kaplan–Meier curves (**E**). (**F**) MCA205 fibrosarcoma-bearing mice received three *i.p.* injections of Sol, Nav, or Ven at day 7 (when palpable MCA205 tumors appear), day 9, and day 11. Subsequent treatments with αPD-1, αCTLA-4, or αIso were applied. (**G-I**) The MCA205 fibrosarcoma growth was monitored and expressed as tumor surface. Statistical significance was calculated by means of the type II ANOVA for tumor growth curves or logrank test for the survival curves. P-values indicate statistical significance.
