# Supplementary material for: BCL2 inhibition reveals a dendritic cell-specific immune checkpoint that controls tumor immunosurveillance
Source: Cancer Discov. Author manuscript; Available in PMC 2023 Nov 1. (PMC7615270; doi:10.1158/2159-8290.CD-22-1338)
Supplement: Figure S6 [file EMS187151-supplement-Figure_S6.pdf]

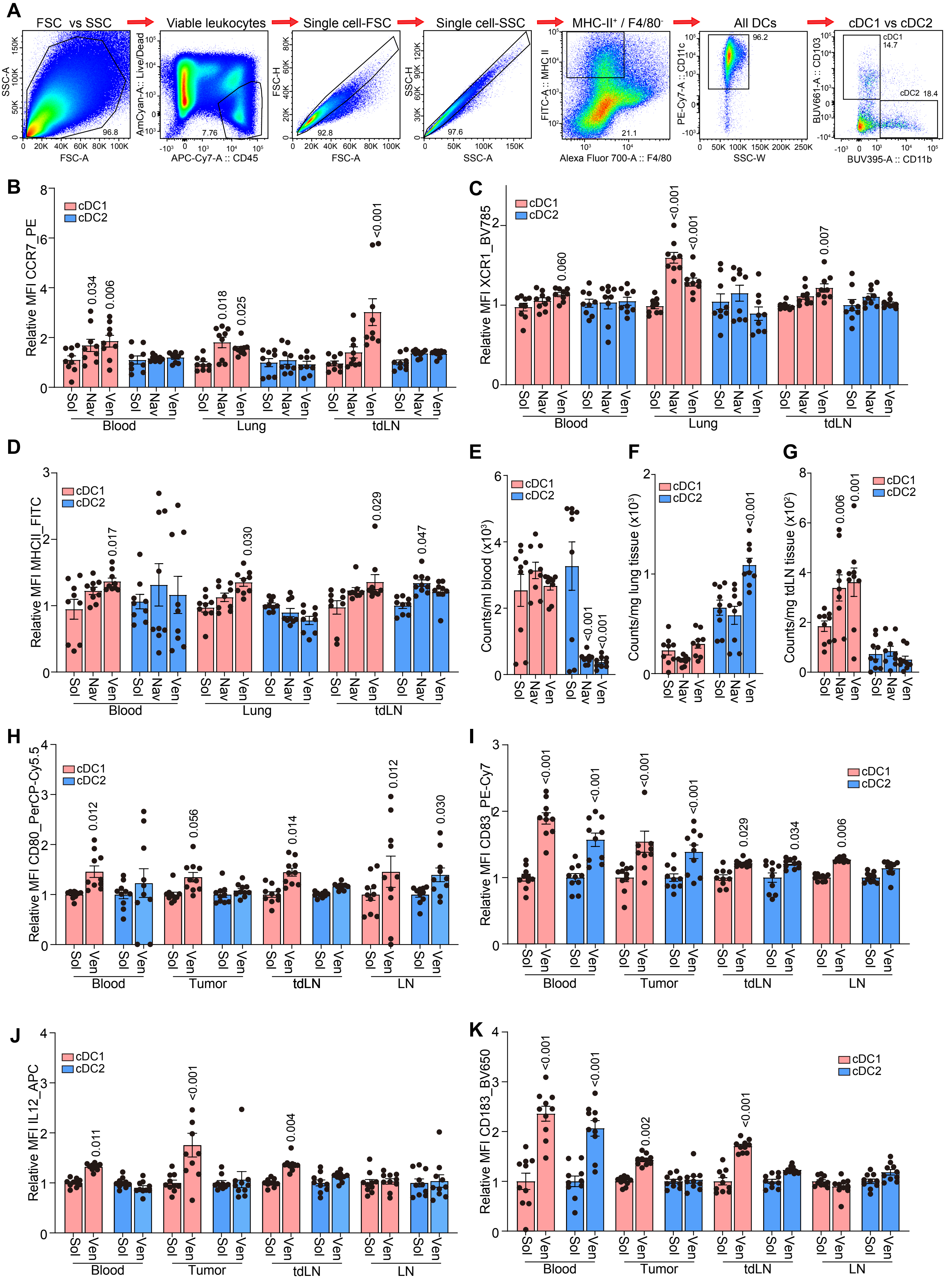

A
FSC vs SSC
Viable leukocytes
Single cell-FSC
Single cell-SSC
MHC-II+ / F4/80-
All DCs
cDC1 vs cDC2
B
C
D
E
F
G
H
I
J
K
cDC1
cDC2
Blood
Lung
tdLN
Tumor
LN
Sol
Nav
Ven
Relative MFI CCR7_PE
Relative MFI XCR1_BV785
Relative MFI MHCII_FITC
Counts/ml blood (x10³)
Counts/mg lung tissue (x10³)
Counts/mg tdLN tissue (x10²)
Relative MFI CD80_PerCP-Cy5.5
Relative MFI CD83_PE-Cy7
Relative MFI IL12_APC
Relative MFI CD183_BV650

Figure S6

Supplementary Figure S6. Impact of Bcl2 inhibitors on DCs *in vivo*. (**A**) Gating strategy for the identification of total DCs (MHC-II^{+} CD11c^{+} F4/80^{-}) among viable leukocytes (CD45^{+} Live/dead^{-}) and the two types of conventional DCs (cDC) among total DCs. (**B-G**) Orthotopic TC1 lung cancer-bearing mice received two *intraperitoneal* (*i.p.*) injections of solvent (Sol), navitoclax (Nav), or venetoclax (Ven) at day 0 (when bioluminescence detectable lung cancers appeared) and day 2. The blood, lung, and lung cancer-draining mediastinal lymph nodes (tdLN) were harvested at day 4 and dissociated into single cell suspensions for multiplex immunostaining and flow cytometric analysis of DCs. The mean fluorescence intensity (MFI) of indicated markers on the type I conventional DCs (cDC1, defined as CD103^{+}CD11b^{-} among DCs) as well as cDC2 cells (CD103^{-}CD11b^{+}) were normalized to the average value of the Sol condition (**B-D**), and the absolute counts of those DC subtypes were calculated based on sampled blood volume and tissue weights (**E-G**), which are depicted as scattered dot plots (n=9 animals/group). (**H-K**) Similar analyses were applied to blood, tumor, tumor-draining and non-tumor-draining inguinal LN from Ven-treated MCA205 fibrosarcoma-bearing mice (n=10 animals/group). Statistical significance was calculated using one-way ANOVA test with Dunnett's multiple comparisons, as compared to Sol. P-values are labelled in the figure to indicate statistical significance.
