# Supplementary material for: BCL2 inhibition reveals a dendritic cell-specific immune checkpoint that controls tumor immunosurveillance
Source: Cancer Discov. Author manuscript; Available in PMC 2023 Nov 1. (PMC7615270; doi:10.1158/2159-8290.CD-22-1338)
Supplement: Figure S7 [file EMS187151-supplement-Figure_S7.pdf]

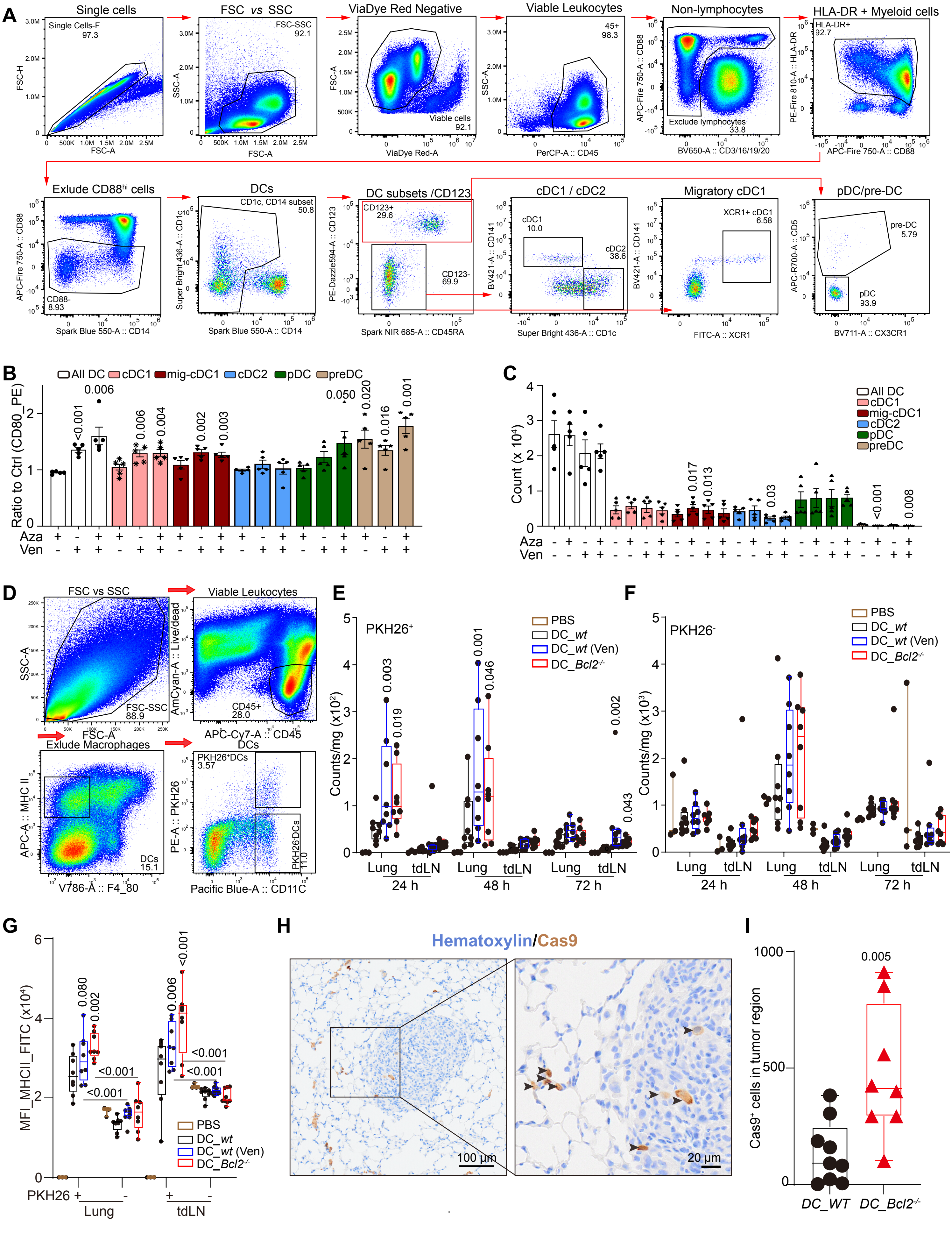
A
Single cells
FSC vs SSC
ViaDye Red Negative
Viable Leukocytes
Non-lymphocytes
HLA-DR + Myeloid cells
Exlude CD88hi cells
DCs
DC subsets /CD123
cDC1 / cDC2
Migratory cDC1
pDC/pre-DC
B
All DC cDC1 mig-cDC1 cDC2 pDC preDC
Ratio to Ctrl (CD80_PE)
Aza
Ven
C
Count (x 10^4)
D
FSC vs SSC
Viable Leukocytes
Exlude Macrophages
DCs
E
PKH26+
Counts/mg (x10^2)
PBS
DC_wt
DC_wt (Ven)
DC_Bcl2-/-
Lung
tdLN
24 h
48 h
72 h
F
PKH26-
Counts/mg (x10^3)
G
MFI_MHCII_FITC (x10^4)
PKH26
Lung
tdLN
H
Hematoxylin/Cas9
100 µm
20 µm
I
Cas9+ cells in tumor region
DC_WT
DC_Bcl2-/-

Figure S7

Supplementary Figure S7. Activation of human peripheral DCs and the migration of adoptively injected de-iniDCs in mouse (A) Gating strategy for the identification of total DCs among viable leukocytes (CD45^{+} Live/dead^{-}) and different subsets of human peripheral DCs. (**B,C**) PBMCs from healthy donors were treated *in vitro* with Ven, Aza, or their combinations and was subjected to multiplex immunostaining and to quantify the expression of distinct DC activation related markers on the surface of DC subpopulations. The ratio of CD80_MFI (mono or combined treatment versus DMSO control) on different DC subtypes, as well as the absolute count of those DCs from individual healthy donors are depicted as scattered dot plots (n=5 donors). A paired t test was performed to calculate p values. (**D-G**) Survival and migration of injected de-iniDCs in mouse. Wild type (wt) de-iniDCs, Ven-treated wt de-iniDCs, or *Bcl2*$^{-/-}$ de-iniDCs were incubated with the lysate of TC1 cells, and stained with a PKH26 red fluorescence dye before intravenously injected into the TC1 lung-cancer bearing mice. The lung and tumor-draining mediastinal lymph nodes (tdLN) were collected at the indicated timepoints and digested to single-cell suspension for multiplex immunostaining. The gating strategy for identifying injected de-iniDCs (PKH26^{+}) and mouse DCs (PKH26^{-}) is illustrated in panel (**D**). Absolute counts of DCs were calculated based on sampled tissue weight (**E, F**). The MFI of MHC-II on those DCs was quantified to indicate their maturation status (**G**). Results and are reported as box plots (mean ± SEM, n=8 animals/group), 3 animals without DC infusion are used as assay controls. Statistical significance was calculated using one-way ANOVA test with Dunnett's multiple comparisons, as compared to the WT DC group or as indicated in panel **G**. P-values are labelled in the figure to indicate statistical significance. (**H,I**) Following the same procedure but without PKH26 staining, the TC1 cancer-bearing lungs were excised at 72 hours after DC injection and perfused and fixed with 4% PFA for immunohistochemistry. De-iniDC_Cas9 cells were detected by immunohistochemistry using an antibody specific for Cas9 in tumor regions counterstained with hematoxylin (**H**). Cas9^{+} cells in the tumor region were counted using QuPath, and their absolute numbers are reported as box plots. Statistical significance was calculated by means of an unpaired *t*-test (**I**).
