# Supplementary material for: BCL2 inhibition reveals a dendritic cell-specific immune checkpoint that controls tumor immunosurveillance
Source: Cancer Discov. Author manuscript; Available in PMC 2023 Nov 1. (PMC7615270; doi:10.1158/2159-8290.CD-22-1338)
Supplement: Figure S8 [file EMS187151-supplement-Figure_S8.pdf]

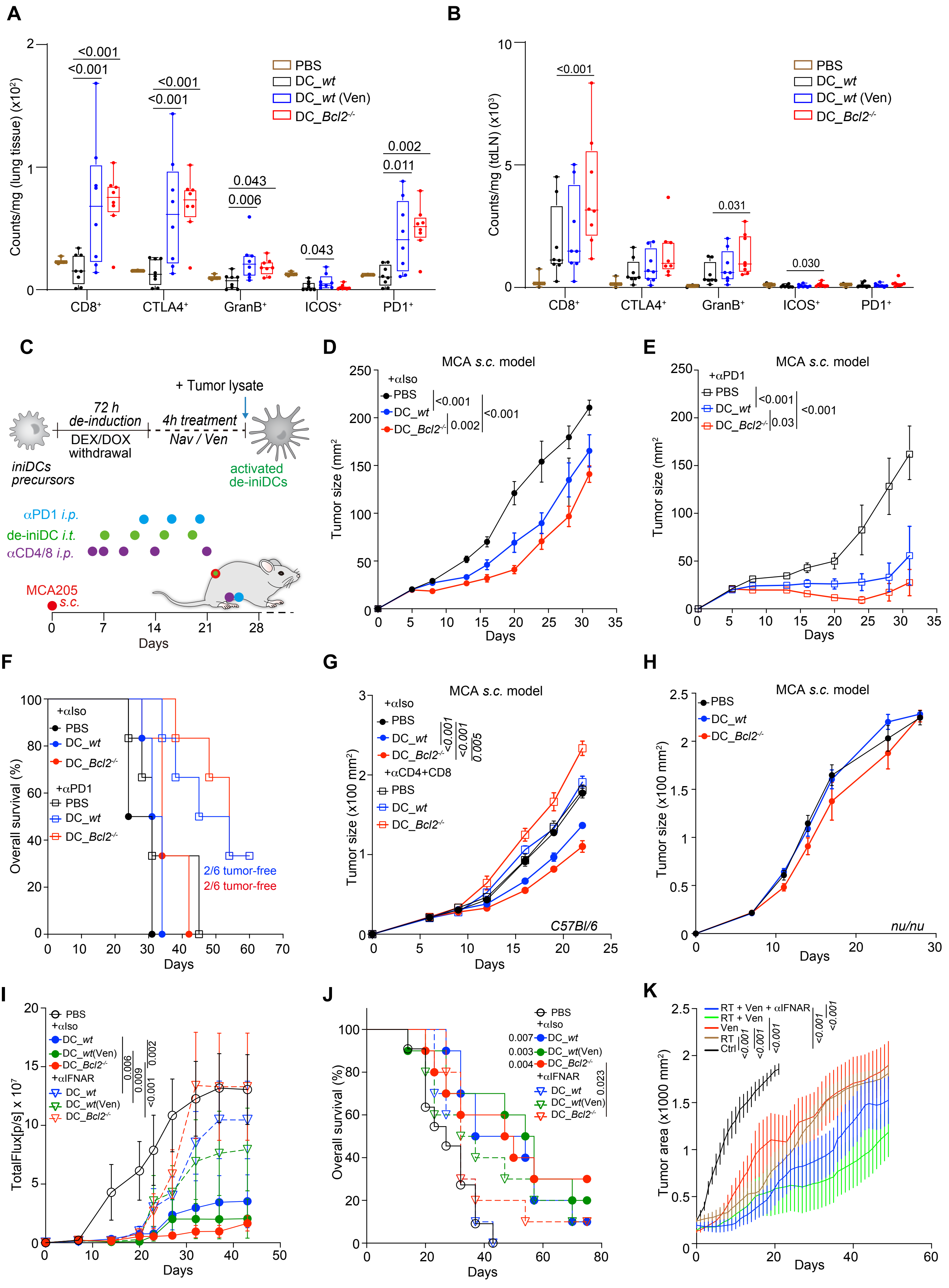
A
B
C
D
E
F
G
H
I
J
K
PBS
DC_wt
DC_wt (Ven)
DC_Bcl2-/-
Counts/mg (lung tissue) (x10²)
Counts/mg (tdLN) (x10³)
CD8⁺
CTLA4⁺
GranB⁺
ICOS⁺
PD1⁺
+ Tumor lysate
72 h de-induction
DEX/DOX withdrawal
4h treatment
Nav / Ven
iniDCs precursors
activated de-iniDCs
αPD1 i.p.
de-iniDC i.t.
αCD4/8 i.p.
MCA205 s.c.
Days
MCA s.c. model
+αIso
+αPD1
Tumor size (mm²)
Overall survival (%)
2/6 tumor-free
2/6 tumor-free
+αCD4+CD8
Tumor size (x100 mm²)
C57Bl/6
nu/nu
TotalFlux[p/s] x 10⁷
+αIFNAR
DC_wt(Ven)
RT + Ven + αIFNAR
RT + Ven
Ven
RT
Ctrl
Tumor area (x1000 mm²)

Figure S8

**Supplementary Figure S8. Adoptively transferred de-iniDCs induce T cell infiltration and suppress tumor growth. (A,B)** Wild type (wt) de-iniDCs, venetoclax (Ven)-treated wt de-iniDCs, or $Bcl2^{-/-}$ de-iniDCs were incubated with the lysate of TC1 cells, and intravenously (*i.v.*) injected into the TC1 lung-cancer bearing mice. The lung and tumor-draining mediastinal lymph nodes (tdLN) were collected 48 hours after and digested to single-cell suspension for multiplex immunostaining of $CD8^+$ T cell related markers. The absolute number of all $CD8^+$ T cells, $CTLA4^+$, granzyme $B^+$ ($GranB^+$), $ICOS^+$, or $PD1^+$ CD8-T cells were calculated and reported as box plots (means ± SEM, n=8 animals/group), 3 animals without DC infusion are used as assay controls. Statistical significance was calculated using one-way ANOVA test with Dunnett's multiple comparisons, as compared to WT DC group. For therapeutic vaccination, WT or *$Bcl2^{-/-}$* de-iniDCs were activated with the lysate of corresponding cancer cells and in some cases, pretreated for 4 h with Ven before activation (**C**). Once the MCA205 tumors became palpable (approximately around day 7), the animals were treated with 4 intratumoral (*i.t.*) injections of de-iniDCs or equivalent volume of PBS, with or without combining neutralizing antibodies to PD-1(αPD-1) or CD4 and CD8 (αCD4/CD8) as illustrated in the scheme (**C**). Tumor size was regularly measured and calculated as surface area. The tumor growth curves including combinations with αPD-1 are reported in **D and E** (mean ± SEM, n = 6 mice/group). The percentage of overall survival is reported in (**F**). The tumor growth curves including combinations with αCD4/CD8 are reported in **G** (mean ± SEM, n = 6 mice/group). The tumor growth curves generated from nude mice (*nu/nu*) with the adoptive DC transfer are reported **H** (mean ± SEM, n = 6 mice/group). (**I,J**) The activated de-iniDCs were further incubated with an monoclonal antibody to IFNAR (αIFNAR) or equal quantities of isotype control antibody (αIso) before *i.v.* injected to TC1 lung-cancer bearing mice. Control mice were *i.v.* injected with only equal volume of PBS. Bioluminescence images were quantified as total flux to indicate tumor size and is reported as tumor growth curves (**I,** mean ± SEM, n = 10 animals/group). Animal survival over time is reported in **J**. (**K**) M/D-driven mammary tumors were randomized to receive vehicle (Ctrl), radiotherapy administered as 10Gy x3 (RT), Ven, RT combined with Ven or RT combined with Ven in the context of IFNAR1 depletion. Tumor areas were monitored until day 50 after treatment initiation. Statistical significance was calculated by means of the type II ANOVA for tumor growth curves, or logrank test for survival curves. P-values are labelled in the figure to indicate statistical significance.
