# Supplementary material for: BCL2 inhibition reveals a dendritic cell-specific immune checkpoint that controls tumor immunosurveillance
Source: Cancer Discov. Author manuscript; Available in PMC 2023 Nov 1. (PMC7615270; doi:10.1158/2159-8290.CD-22-1338)
Supplement: Figure S9 [file EMS187151-supplement-Figure_S9.pdf]

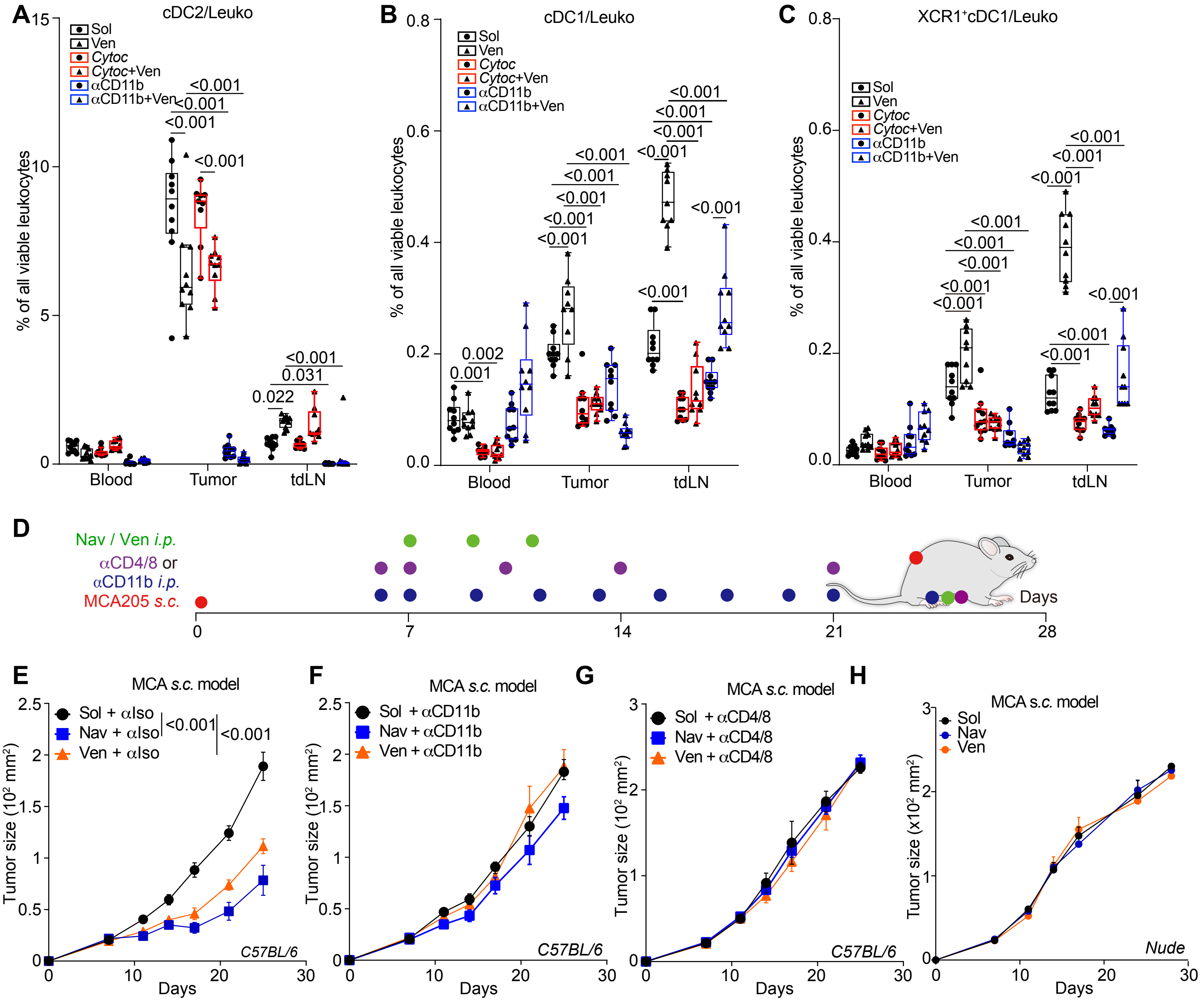
A
cDC2/Leuko
Sol
Ven
Cytoc
Cytoc+Ven
αCD11b
αCD11b+Ven
% of all viable leukocytes
Blood
Tumor
tdLN
B
cDC1/Leuko
C
XCR1+cDC1/Leuko
D
Nav / Ven i.p.
αCD4/8 or
αCD11b i.p.
MCA205 s.c.
Days
E
MCA s.c. model
Sol + αIso
Nav + αIso
Ven + αIso
Tumor size (10² mm²)
C57BL/6
F
Sol + αCD11b
Nav + αCD11b
Ven + αCD11b
G
Sol + αCD4/8
Nav + αCD4/8
Ven + αCD4/8
H
Sol
Nav
Ven
Tumor size (x10² mm²)
Nude

Figure S9

Supplementary Figure S9. Systemic administration of Bcl2 inhibitors exerts immune-dependent anticancer efficacy on orthotopic fibrosarcoma. Orthotopic MCA205 fibrosarcomas were established by subcutaneous (*s.c.*) injection of MCA205 cells on C57BL/6 mice or nude mice. (**A-C**) Once the tumors became palpable, the mice were treated intravenously (*i.v.*) with cytochrome c (*Cytc*) *vs* PBS, or intraperitoneally (*i.p.*) with CD11b blocking antibody (αCD11b) *vs* isotype control (αIso) at day0, day1, day3; in combination with *i.p.* venetoclax (Ven) *vs* solvent (Sol) at day0 and day2. The blood, tumor, and tumor-draining lymph nodes (tdLN) were harvested at day 4 and dissociated into single cell suspensions for multiplex immunostaining and flow cytometric analysis. The percentage of type II conventional DCs (cDC2, defined as F4/80$^-$ MHC-II$^+$CD11c$^+$CD103$^-$CD11b$^+$ among viable leukocytes, **A**), cDC1 cells (CD10$^+$CD11b$^-$, **B**), as well as migratory XCR1$^+$cDC1 cells (**C**) within all viable leukocytes is depicted as scattered dot plots (n=10 animals/group). Statistical significance was calculated using one-way ANOVA test with Dunnett's multiple comparisons, as comparing between indicated groups. (**D-H**), The MCA205 fibrosarcoma-bearing animals were subjected for the treated with Sol, navitoclax (Nav), or Ven, at day7 (when tumors become palpable), 9, and 11 with or without combining neutralizing antibodies to CD11b or CD4 and CD8 (αCD4/CD8) as illustrated in the scheme (**D**). Tumor size was regularly measured and calculated as surface area, which are reported as tumor growth curves (**E-H**, mean ± SEM). Statistical significance was calculated by means of the type II ANOVA, n = 6 mice/group.
