# Supplementary material for: BCL2 inhibition reveals a dendritic cell-specific immune checkpoint that controls tumor immunosurveillance
Source: Cancer Discov. Author manuscript; Available in PMC 2023 Nov 1. (PMC7615270; doi:10.1158/2159-8290.CD-22-1338)
Supplement: Figure S10 [file EMS187151-supplement-Figure_S10.pdf]

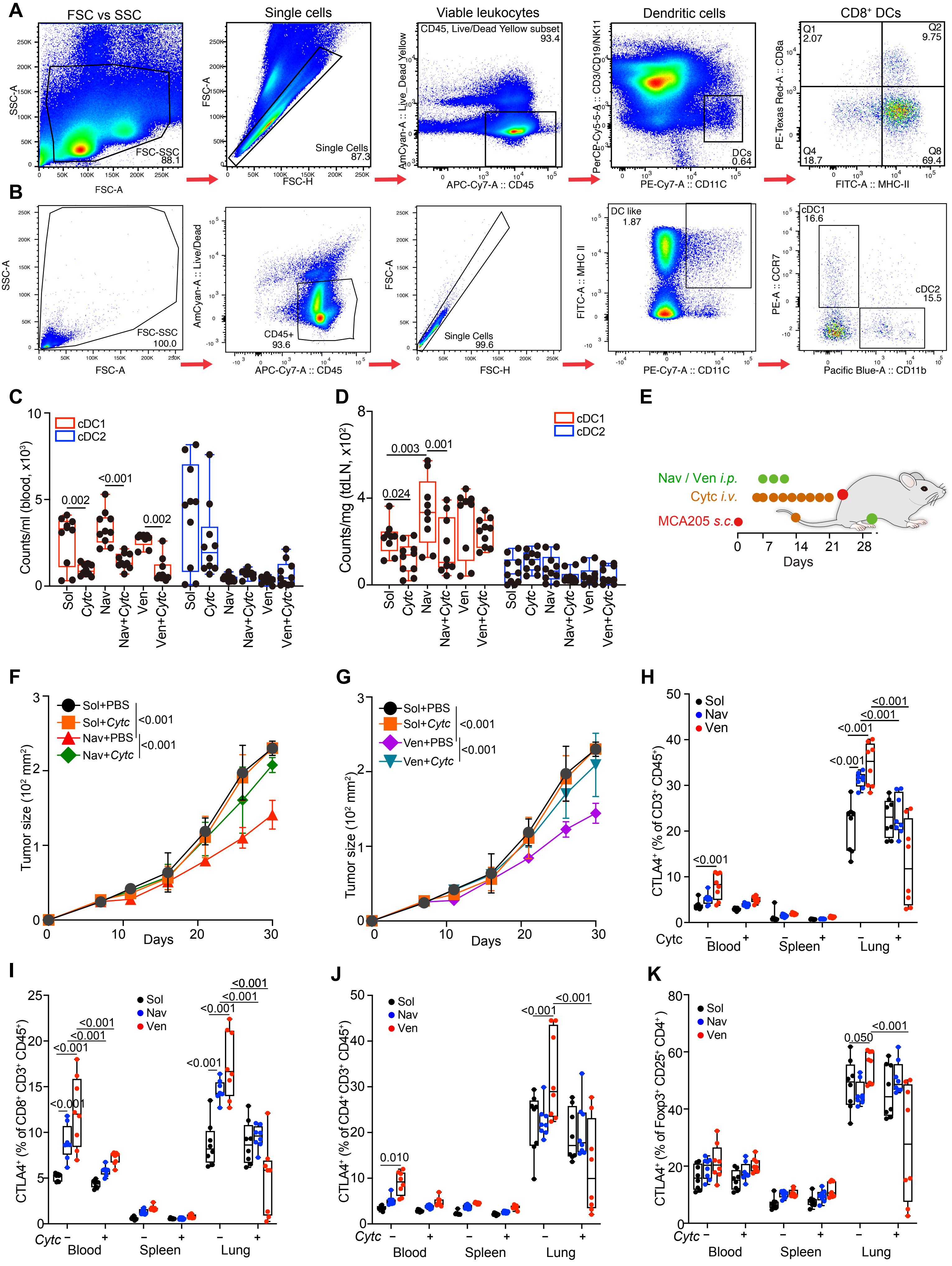
A
FSC vs SSC
Single cells
Viable leukocytes
Dendritic cells
CD8⁺ DCs
FSC-SSC
88.1
Single Cells
87.3
CD45, Live/Dead Yellow subset
93.4
DCs
0.64
Q1
2.07
Q2
9.75
Q4
18.7
Q8
69.4
FSC-A
SSC-A
FSC-H
AmCyan-A :: Live_Dead Yellow
APC-Cy7-A :: CD45
PerCP-Cy5-5-A :: CD3/CD19/NK11
PE-Cy7-A :: CD11C
PE-Texas Red-A :: CD8a
FITC-A :: MHC-II
B
FSC-SSC
100.0
AmCyan-A :: Live/Dead
CD45+
93.6
Single Cells
99.6
DC like
1.87
FITC-A :: MHC II
PE-A :: CCR7
cDC1
16.6
cDC2
15.5
Pacific Blue-A :: CD11b
C
cDC1
cDC2
Counts/ml (blood, x10³)
0.002
<0.001
0.002
Sol
Cytc
Nav
Nav+Cytc
Ven
Ven+Cytc
D
Counts/mg (tdLN, x10²)
0.003
0.001
0.024
E
Nav / Ven i.p.
Cytc i.v.
MCA205 s.c.
Days
F
Sol+PBS
Sol+Cytc
Nav+PBS
Nav+Cytc
<0.001
<0.001
Tumor size (10² mm²)
Days
G
Sol+PBS
Sol+Cytc
Ven+PBS
Ven+Cytc
<0.001
<0.001
H
Sol
Nav
Ven
CTLA4⁺ (% of CD3⁺ CD45⁺)
<0.001
Cytc
Blood
Spleen
Lung
I
CTLA4⁺ (% of CD8⁺ CD3⁺ CD45⁺)
J
CTLA4⁺ (% of CD4⁺ CD3⁺ CD45⁺)
0.010
K
CTLA4⁺ (% of Foxp3⁺ CD25⁺ CD4⁺)
0.050

Figure S10

Supplementary Figure S10. Identification of type I conventional DCs (cDC1) and their depletion by cytochrome *c*. (A) Gating strategy for the identification of cDC1 in splenocytes from bone marrow reconstituted mice. (**B-E**) Orthotopic TC1 lung tumor-bearing mice were intravenously (*i.v.*) treated with cytochrome C (Cyt c) starting at first detection and maintained every other day, and then followed by intraperitoneal (*i.p.*) treatment of solvent (Sol), navitoclax (Nav), or venetoclax (Ven). Blood and tumor-draining lymph nodes (tdLN) were harvested 4 days later and were dissociated into single cell suspensions for multiplex immunostaining and flow cytometric analyses. Absolute numbers cDC1 (defined as $CCR7^{+}CD11b^{-}$ among all DCs, which are Live/dead^{-} $CD45^{+}$ MHC-$II^{hi}CD11c^{hi}$, **B**) and cDC2 ($CCR7^{-}CD11b^{+}$) in blood and tdLN are reported as box plots (**C,D**). Statistical significance was calculated by means of a one-way ANOVA test with Dunnett's multiple comparisons. **(E-G)** Orthotopic MCA205 fibrosarcomas were established by subcutaneous (*s.c.*) injection of MCA205 cells on C57BL/6 mice at day 0. Once the tumors became palpable (~ day 6), the animals were pre-treated with 5 mg/mouse of Cytc or PBS, every other day, by *i.v.* injection maintained for two weeks, followed by *i.p.* treatment with either Sol, Nav, or Ven, as depicted in the scheme (**E**). Tumor size was regularly measured and calculated as surface area, which are reported as tumor growth curves (**F, G**, mean ± SEM, n=7 animals/group). Statistical analysis was performed by type II ANOVA. (**H-K**) Orthotopic TC1 lung tumor-bearing mice were treated as described above. Spleen, lung, and blood were harvested 7 days later and were dissociated into single cell suspensions for multiplex immunostaining and flow cytometric analysis. The percentage of $CTLA4^{+}$ cells in total T cells (**H**), $CD8^{+}$ T cells (**I**), $CD4^{+}$ T cells (**J**), as well as Tregs (defined as $CD4^{+}FOXP3^{+}CD25^{+}$, **K**) among total T cells were evaluated with FlowJo and depicted as dot plots (n=8 animals/group). Statistical significance was calculated using the one-way ANOVA test.
