# Supplementary material for: BCL2 inhibition reveals a dendritic cell-specific immune checkpoint that controls tumor immunosurveillance
Source: Cancer Discov. Author manuscript; Available in PMC 2023 Nov 1. (PMC7615270; doi:10.1158/2159-8290.CD-22-1338)
Supplement: Supplementary table legends [file EMS187151-supplement-Supplementary_table_legends.pdf]

# Legends for Supplementary Tables

**Supplementary Table S1. List of genes in the genome-wide CRISPR library.**

This table is related to Figure 1, and contains the list of genes covered by the genome-wide CRISPR library and statistical values generated with the MAGeCK analysis tool (tab "All genes"). Top enriched genes in the selected cells with a gain-of-function phenotype are listed in the tab "715 hits". *pos|score* represents the robust ranking aggregation (RRA) value of this gene in positive selection; *pos|lfc* represents the log fold change of this gene in positive selection.

**Supplementary Table S2. List of genes in the arrayed CRISPR screening.**

This table is related to Figure 2, and contains the list of genes in the arrayed CRISPR screening, the log2 fold change (logfc, as compared with the non-target control crRNA) of IL2 production, p-values, and the annotation of their link to the enriched druggable pathways. The second tab contains top genes the KO of which significantly increased antigen presentation by de-iniDCs in the screening reported in Fig. 2A and B.

**Supplementary Table S3. List of significantly upregulated genes in the bulk RNA Seq.**

This table is related to Figure 2, and contains a list of genes that are more than averagely 1.5-fold upregulated in de-iniDCs by *Bcl2*-KO and venetoclax treatment. Columns include the average fold change (FC) of *Bcl2-KO* vs WT and Venetoclax-treated vs untreated de- iniDCs (FC mean); FC and p-values of Bcl2-KO vs WT (FC Bcl2-KO, p-values Bcl2-KO) and Venetoclax-treated vs untreated de- iniDCs (FC Ven, p-values Ven), as well as the reference indicating the link of each gene to the Type 1 interferon pathway.

**Supplementary Table S4. List of primers used for quantitative real-time PCR.**
